# Molecular Mechanisms of Dopaminergic Neuron Degeneration in Parkinson’s Disease: A Comprehensive Review

**DOI:** 10.2174/011570159X446831260303200516

**Published:** 2026-04-07

**Authors:** Md Abubakar, Shahnaz Alom, Farak Ali, Sheikh Rezzak Ali, Krishnendu Adhikary, Sarika, Prakash Kumar Gupta, Eswara Rao Puppala, Krishna Murti, Sachchida Nand Rai, Nitesh Kumar

**Affiliations:** 1 Department of Pharmacology and Toxicology, National Institution of Pharmaceutical Education and Research, Hajipur, Vaishali, Bihar, 844102, India;; 2 Department of Pharmaceutical Sciences, Girijananda Chowdhury Institute of Pharmaceutical Science, Girijananda Chowdhury University-Tezpur Campus, Tezpur, Assam, 784501, India;; 3 Department of Pharmaceutical Sciences, Faculty of Science and Engineering, Dibrugarh University, Dibrugarh, Assam, 786004, India;; 4 Department of Medical Laboratory Technology, Paramedical College Durgapur, West Bengal, 713212, India;; 5 School of Biological and Environmental Sciences, Shoolini University of Biotechnology and Management Sciences, Solan, Himachal Pradesh, 173229, India;; 6 Department of Pharmacy, Central University of South Bihar, Gaya NH-120, Gaya Panchanpur Road, Village - Karhara, Post - Fatehpur, Gaya, Bihar, 824236, India;; 7 Department of Surgery, School of Medicine, Washington University in St. Louis, St. Louis, Missouri, 63110, USA;; 8 Department of Pharmacy Practice, National Institution of Pharmaceutical Education and Research, Hajipur, Vaishali, Bihar, 844102, India;; 9 Centre of Experimental Medicine and Surgery, Institute of Medical Sciences, Banaras Hindu University, Varanasi, 221005, India;; 10 University Centre for Research & Development (UCRD), Chandigarh University, Mohali, Punjab, 140413, India

**Keywords:** Dopamine, endoplasmic reticulum, lewy body, mitochondria, neurodegeneration, Parkinson’s disease, α-synuclein

## Abstract

Parkinson's disease (PD) is a neurological condition that starts with the degeneration of neurons. Neurons play a crucial role in producing dopamine (DA), a type of neurotransmitter that primarily regulates bodily functions such as motor control, posture, motivation, reward, pleasure, cognition, and memory. Other variables that contribute to the disorder include the buildup of Lewy bodies and Lewy neurites, which are composed of increased α-synuclein (α-syn). Depletion of DA in the striatal area and the death of DA-producing neurons are often considered the basis for the motor impairments seen in PD. In addition, both genetic and environmental factors may play a role in PD etiology; specifically, genetic variations and exposure to toxins may contribute to the development of brain lesions. The article aims to outline the current state of knowledge on the dopaminergic pathway and how PD affects DA homeostasis. Various molecular mechanisms are involved in the pathogenesis of PD, including α-syn aggregation, lysosomal and chaperone-mediated autophagy, mitochondrial dysfunction, and abnormal regulation of calcium homeostasis. Intrinsic and extrinsic caspase-mediated apoptosis, autophagic cell death, and ferroptosis are also involved in neurodegeneration that often leads to PD. The occurrence of PD can be controlled by the inclusion of antioxidants, such as mitoquinone, which inhibit mitochondrial oxidative damage, as well as modulation of autophagy, proteostasis, gene therapy, and its editing, and stem cell regeneration. Diverse mechanistic pathogenesis and genetic variations make PD a complicated disease to tackle. Potential treatment approaches, such as modulating autophagy-lysosomal pathways and protecting mitochondria, may be better understood with deeper insight into these mechanisms. We conclude by highlighting current and upcoming gene and cell therapies.

## 1 INTRODUCTION

As individuals become older, they are more likely to develop Parkinson's disease (PD), a neurodegenerative disorder characterized by the progressive and localized loss of dopamine (DA) neural networks in the SN, an area critical for motor function in the brain. As a result of this neuronal loss, DA levels in the striatum significantly decrease, disrupting the regulation of basal ganglia circuits that control voluntary movement. Clinically, PD manifests through cardinal motor symptoms- bradykinesia, resting tremor, muscular rigidity, and postural instability- besides non-motor features, including sleep disorders, constipation, depression, hyposmia, and cognitive decline [1-4]. Recent estimates suggest that PD affects more than 8.5 million individuals globally, underscoring the urgency of elucidating its molecular underpinnings [5]. Although the disease has traditionally been viewed through the lens of DA deficiency, current understanding emphasizes that PD pathophysiology involves an interplay among mitochondrial dysfunction, oxidative stress, α-synuclein (α-syn) aggregation, neuroinflammation, impaired autophagy, and genetic susceptibility [6-8]. The term "oxidative stress" describes a state in which the production of reactive oxygen species (ROS) outstrips the antioxidant capacity of cells, either because the antioxidant system is malfunctioning or because ROS creation is increased. Neurodegenerative disorders such as AD and PD are among the many human illnesses that may result from biochemical changes in these macromolecular components [9].

Environmental exposures such as metals (*e.g*., manganese) and pesticides (*e.g*., rotenone, paraquat) have also been implicated in neuronal injury, suggesting a strong link between toxin exposure and disease progression in susceptible individuals [10, 11]. The presence of Lewy bodies (LBs), intracellular inclusions primarily composed of aggregated α-syn and ubiquitin, is a key neuropathological characteristic of PD in surviving neurons. However, the mechanisms underlying DA neuron degeneration remain incompletely understood. It is increasingly accepted that PD is not confined to the substantia nigra (SN) alone, but represents a systemic disorder with pathologies extending to the enteric nervous system, olfactory bulb, and even the peripheral immune system [12]. These insights have broadened the conceptual framework of PD from a localized brain disorder to a multisystem disease. Despite decades of research, no treatment currently halts or reverses the progression of the disease. Most therapies, such as levodopa and DA agonists, provide symptomatic relief but do not address the underlying causes of DA cell death. Consequently, there is growing attention towards developing neuroprotective approaches that target early molecular events in the pathogenesis of PD, including mitochondrial repair, restoration of redox balance, inhibition of α-syn aggregation, and modulation of inflammatory responses [13].

To better understand DA neuron loss in PD, this review aims to elucidate the primary mechanisms underlying its occurrence. Various interrelated mechanisms have been investigated, including DA metabolism, mitochondrial malfunction, α-syn misfolding, calcium dysregulation, immunological activation, and abnormalities in DA release. We have discussed various neuroprotective and cell-based therapeutics, as well as management techniques. To help guide the creation of new treatments and the identification of biomarkers, we are striving to provide an integrated viewpoint. We reviewed the scientific literature using PubMed and other supplementary resources, including ScienceDirect, Google Scholar, DOAJ, Web of Science, Wiley, and Scopus. Journals published between 2005 and 2025 were selected for the current review work. The journal has been chosen based on the description of the required data, as defined by the searched keywords and Boolean operators such as "OR" and "AND", to refine the studies. The searched keywords for references that encompass the results of a beforehand carried out systematic search includes “pathophysiology AND/OR PD,” “DA metabolism,” “DA pathway,” “DA release defects,” “synucleinopathy and PD,” “mitochondrial dysfunction AND/OR PD,” “ER stress AND/OR PD,” “Ca^2+^ dysregulation AND/OR PD,” “mechanism of neurodegeneration AND/OR PD,” “genetic mutation AND/OR PD,” “environmental toxin AND/OR PD,” “translational model AND/OR PD,” “neuroprotective strategies AND/OR PD,” “autophagy modulation AND/OR PD,” and “stem cell-based therapies AND/OR PD.” Additional search engines, such as “DA pathway AND/OR PD,” “autophagy dysfunction AND/OR PD,” “lysosomal dysfunction AND/OR PD,” “environmental toxins AND/OR PD,” “antioxidant AND/OR PD,” “anti-inflammatory drugs AND/OR PD,” “gene therapy AND/OR PD,” “CRISPR-Cas9 AND/OR PD,” “RNA-based therapies AND/OR PD,” were utilized in the development of this article.

### 1.1 Epidemiology of PD

The burden of PD is steadily growing due to an aging global population and extended life expectancy. The PD affects more than 10 million people worldwide, making it the second most common neurodegenerative disorder (NDD) after Alzheimer's disease (AD). Its prevalence is approximately 1% in individuals aged 60 and older and up to 4% in those aged 80 and older [14]. The disease typically begins in the sixth or seventh decade of life, although early-onset forms can present before the age of 50, often with a genetic basis [15]. Epidemiological studies have shown a slightly higher prevalence in men compared to women, with ratios ranging from 1.2:1 to 1.5:1. This disparity may be linked to estrogen’s neuroprotective effects, occupational exposures, or sex-related genetic differences [16]. There are significant regional variations in the epidemiology of PD. About 49 per 1,000 people in Sub-Saharan Africa have the lowest count, but Latin America and East Asia have much higher rates, about 600 per 1,000. The disease is more common in urban than rural areas. In the US, white groups have a two times greater chance than black populations [17, 18]. Genetic factors account for approximately 5-10% of all cases, with mutations in SNCA, PINK1, LRRK2, PARK (1 and 7), and GBA genes playing significant roles. These mutations often lead to early-onset or atypical presentations [19]. Environmental exposures also contribute to disease risk. Chronic exposure to pesticides such as paraquat and maneb, industrial solvents such as trichloroethylene, and heavy metals such as manganese has been associated with an increased risk of PD [20]. Conversely, protective factors such as caffeine consumption, nicotine use, and physical activity have shown consistent inverse correlations, although their mechanisms are not fully understood [21-23]. The circumstance is also drawing increasing attention to comorbidities and systemic factors in PD risk. Conditions like diabetes, chronic inflammation, and metabolic syndrome have been linked to increased susceptibility, possibly through shared inflammatory or oxidative pathways [24]. Additionally, rural living and farming occupations are frequently associated with a higher prevalence of PD, likely due to increased exposure to pesticides [25]. The future landscape of PD epidemiology is concerning. Predictions indicate that the prevalence of PD will exceed twofold by the year 2040, particularly in nations with poor or medium incomes where diagnostic and treatment resources are limited [26]. This looming healthcare challenge underscores the importance of early detection, preventive strategies, and equitable access to care.

### 1.2 Introduction to DA

The DA is a key catecholamine neurotransmitter that plays a central role in regulating motor control, cognition, emotion, reward, and endocrine functions. It is synthesized primarily in midbrain regions, particularly the SN and the ventral tegmental area (VTA), from which it projects to the striatum, prefrontal cortex, limbic system, and hypothalamus [27, 28]. The DA system is crucial for maintaining the functional integrity of the basal ganglia, a brain circuit heavily involved in voluntary movement. Disruption of this system, especially the NS pathway, is a hallmark of PD [29]. Tyrosine is the first amino acid used to make DA. The step that slows down the process is the conversion of tyrosine to L-DOPA by the enzyme tyrosine hydroxylase (TH), followed by decarboxylation of L-DOPA by aromatic L-amino acid decarboxylase (AADC), which forms DA. This process is tightly regulated by feedback mechanisms and the availability of cofactors such as tetrahydrobiopterin (BH4) [30]. Once synthesized, DA is stored in synaptic vesicles (SVs) *via* VMAT2 (vesicular monoamine transporter 2) and released into the synaptic cleft upon neuronal stimulation. It binds to DA receptors (D1-D5 subtypes), triggering intracellular signaling cascades that influence postsynaptic activity [31, 32]. While DA itself cannot cross the blood-brain barrier (BBB), the precursor L-DOPA can readily cross the BBB and be transformed into DA inside the brain, increasing its concentration. This is why L-DOPA is used to treat PD. This also underlines the importance of endogenous DA synthesis and turnover within the central nervous system (CNS). In the striatum, DA acts on both D1-like (excitatory) and D2-like (inhibitory) receptors to modulate motor output. DA loss disrupts this balance, leading to the characteristic motor symptoms of PD, such as tremor, rigidity, and slow movement, as shown in Fig. (**1**) [32-36]. Beyond its role in movement, DA is involved in neuroplasticity, memory formation, emotional regulation, and motivational behaviour. The mesolimbic and mesocortical DA pathways are particularly implicated in mood disorders, addiction, and cognitive functions. Thus, DA influence extends far beyond the NS system [37]. In PD, DA loss is accompanied by degeneration of DA neurons and accumulation of α-syn aggregates, leading to both presynaptic and postsynaptic dysfunctions. This disrupts synaptic transmission, weakens compensatory neuronal circuits, and accelerates neurodegeneration [38]. The vulnerability of DA neurons may be attributed to their high metabolic demand, extensive axonal arborization, and intrinsic electrophysiological properties that make them more susceptible to oxidative stress and mitochondrial dysfunction [39]. Understanding the multifaceted roles of DA in the CNS is crucial for interpreting PD pathogenesis and guiding therapeutic strategies, including DA replacement therapies, receptor agonists, and agents that target DA neuron preservation [40].

## 2 DA SYSTEM AND ITS INTERLINK PATHWAYS

### 2.1 Nigrostriatal (NS) Pathway

The NS pathway is one of the principal DA circuits in the brain and plays a critical role in motor control. Originating from the SN, DA neurons project to the dorsal striatum, primarily targeting the caudate nucleus and putamen (Table **1**). This pathway modulates the activity of basal ganglia circuits that coordinate voluntary movement. In PD, the hallmark pathological feature is the deterioration of DA neurons over time, resulting in a significant decline in striatal DA levels, which leads to motor dysfunction, including bradykinesia, stiffness, resting tremor, and rigidity (Table **1**) [8, 41]. Neuroimaging studies using positron emission tomography (PET) and single-photon emission computed tomography (SPECT) have consistently demonstrated reduced striatal dopamine transporter binding in patients with PD, supporting previous findings and indicating disease progression [42]. Moreover, postmortem analyses reveal LBs, composed predominantly of misfolded α-syn, within SN neurons, implicating synucleinopathies as a central mechanism of NS degeneration [41]. Oxidative stress, mitochondrial dysfunction, and impaired autophagy further exacerbate neuronal loss in this pathway (Table **2**) [43, 44]. Understanding the vulnerability of NS neurons has therapeutic implications. DA therapies, such as L-DOPA and DA agonists, aim to compensate for DA deficiency, although they do not halt disease progression. Neuroprotective strategies are being explored to preserve the integrity of this critical motor pathway.

### 2.2 Mesocortical Pathway

This pathway projects DA fibres from the VTA to the prefrontal cortex, which is primarily involved in cognitive control, motivation, and emotional regulation. Dysfunction in this circuit has been implicated in the non-motor PD symptoms such as cognitive impairment, depression, and executive dysfunction (Table **1**) [27, 45]. Unlike the NS pathway, the mesocortical tract is relatively spared in early PD, though subtle deficits can appear in prodromal stages. The DA transmission in this circuit modulates working memory and decision-making, processes governed by the dorsolateral prefrontal cortex. As PD advances, mesocortical dysfunction may contribute to the development of PD dementia (PDD), a debilitating cognitive decline in late stages [46]. Neurochemical studies show that DA levels and D1 receptor density are notably reduced in the frontal cortex of PD patients. These alterations impair cortical plasticity and reduce neural efficiency [47]. Pharmacological modulation using monoamine oxidase-B (MAO-B) inhibitors and DA reuptake blockers has shown partial cognitive benefits, although the risk-benefit balance is still under investigation [48]. Thus, while not a primary target of PD pathology, mesocortical impairment is a crucial contributor to the disease's heterogeneous symptomatology.

### 2.3 Mesolimbic Pathway

The mesolimbic pathway is a DA circuit projecting from the VTA to limbic structures such as the hippocampus, nucleus accumbens, and amygdala. This pathway is central to the regulation of reward, reinforcement learning, emotional responses, and motivation (Table **1**). While traditionally studied in the context of addiction and psychiatric disorders, emerging evidence suggests that dysfunction of the mesolimbic system also contributes to non-motor symptoms in PD, such as anhedonia, apathy, and depression [28, 49]. PD patients often exhibit diminished DA activity in the mesolimbic pathway, which correlates with motivational deficits. Functional imaging studies have shown reduced DA transporter binding in the nucleus accumbens and medial forebrain regions in PD cohorts with high apathy scores [50]. This hypodopaminergic state likely disrupts the mesolimbic feedback loop, which is essential for processing rewarding stimuli and initiating goal-directed behaviour. Additionally, chronic DA therapies, especially DA agonists, may paradoxically overactivate this circuit, leading to impulse control disorders (ICDs) such as pathological gambling and hypersexuality in some PD patients [51]. This biphasic effect highlights the delicate balance required to modulate the mesolimbic system during PD treatment. Hence, targeting this pathway therapeutically requires a nuanced approach that considers both its hypoactivity and hyperreactivity, potentially using receptor-specific agents and behavioural interventions [52].

### 2.4 Tuberoinfundibular Pathway

The tuberoinfundibular pathway is a neuroendocrine DA circuit that extends from the arcuate nucleus of the hypothalamus to the median eminence, where DA is released into the hypophyseal portal system. This DA acts to inhibit prolactin secretion from the anterior pituitary gland (Table **1**). In the context of PD, this pathway is typically preserved, but its importance becomes evident when DA drugs are introduced [53, 54]. DA agonists used in PD treatment can suppress prolactin levels by stimulating D2 receptors, sometimes leading to hypoprolactinemia and associated reproductive or metabolic side effects. Conversely, DA antagonists, such as antipsychotics, may block this pathway and induce hyperprolactinemia [55]. Therefore, monitoring hormonal balance is essential during PD pharmacotherapy, especially in patients with comorbid psychiatric disorders requiring antipsychotic intervention. Neuroendocrine interactions between this pathway and the hypothalamic-pituitary axis highlight the broader physiological implications of DA modulation in PD. Disruptions in circadian rhythm, thermoregulation, and appetite observed in PD may be partially attributed to hypothalamic DA dysfunction, though these associations require further exploration [46, 47]. Understanding the tuberoinfundibular pathway helps distinguish primary disease effects from treatment-related endocrine disturbances and underscores DA's systemic role beyond the motor system.

### 2.5 Direct and Indirect Pathways (PKA/DARPP-32, ERK, and mTORC1)

These pathways of the basal ganglia are central to motor control and are finely regulated by DA signaling. The direct pathway facilitates movement through D1 DA receptor-expressing striatal neurons, whereas the indirect pathway inhibits movement *via* D2 receptor-expressing neurons. Disruption in the balance between these circuits underlies the motor symptoms of PD [35, 56-58]. Activation of the D1 receptor in the direct pathway engages the PKA/DARPP-32 cascade, thereby promoting excitability and motor output. Conversely, D2 receptor activation suppresses cAMP production, reducing the excitability of the indirect pathway. DA depletion in PD diminishes D1 signaling while overactivating the inhibitory indirect path, leading to bradykinesia and rigidity [59-64]. Beyond classical pathways, intracellular cascades such as ERK (extracellular signal-regulated kinase) and mTORC1 (mechanistic target of rapamycin complex 1) are also implicated in synaptic plasticity and DA neurodegeneration [65-67]. Additionally, ERK is activated by signals from both D1 and glutamatergic inputs, influencing gene activity related to adaptability and survival. Dysregulated ERK signaling is observed in L-DOPA-induced dyskinesia [68-71]. mTORC1, a key regulator of cellular growth and autophagy, is upregulated in PD models and may contribute to α-syn accumulation and DA cell stress [72-74]. Therapeutic strategies aimed at modulating these signaling cascades may offer neuroprotective benefits or improve motor outcomes in PD, underscoring the intracellular architecture of direct and indirect pathways as a key focus for drug development.

## 3 DA METABOLISM

DA metabolism is a finely tuned process that balances neurotransmitter activity and degradation to maintain synaptic homeostasis. In DA neurons, DA is a messenger substance responsible for signal transduction. It emerges as a transitional substance between norepinephrine and epinephrine [89, 90]. In catecholamine neurons, DA is produced in two phases. The TH first transforms the amino acid tyrosine into L-DOPA, which is decarboxylated to DA [91]. VMAT2 in the cytoplasm of DA neurons carries and stores the produced DA in vesicles during the resting state, with the help of a vesicular ATPase-based H^+^ gradient. When found on SVs, VMAT2 functions as a stoichiometric antiporter in acidic environments, transporting one monoamine molecule into vesicles and two H^+^ ions out of the vesicles [92]. When neurons are stimulated, DA is transported into the synaptic cleft, facilitating signal transmission. Once DA is released into the synaptic cleft and binds its receptors, it can be either reabsorbed by presynaptic neurons or metabolized by specific enzymes. The primary pathway for DA degradation involves COMT (catechol-O-methyl transferase) and MAO (monoamine oxidase) [93]. Inside the cell, DA is broken down by MAO-B into 3,4-dihydroxyphenylacetic acid (DOPAC), which COMT then changes into homovanillic acid (HVA), the main product of DA breakdown in the brain [94]. Alternatively, DA can first be methylated by COMT to form 3-methoxytyramine (3-MT), which is then oxidized to HVA by MAO. These metabolites are transported out of the brain and excreted in the urine. Abnormal levels of HVA in CSF and plasma have been used as biomarkers of DA activity [95]. In PD, reduced DA availability is not solely due to neuronal death but also involves dysregulated metabolism. Oxidative deamination of DA by MAO-B generates H₂O₂, which in the presence of free iron leads to the formation of •OH through the Fenton reaction. These ROS contribute to oxidative stress, damaging lipids, proteins, and DNA, and thereby accelerating neurodegeneration [96, 97]. Additionally, cytosolic DA that is not sequestered in vesicles can auto-oxidize, forming toxic quinones and semiquinones that modify cellular proteins, including α-syn. The DA-quinone adducts alter protein structure and function, impair proteasomal degradation, and facilitate aggregation of misfolded proteins- a key feature in PD pathology [98]. The VMAT2 plays a critical role in protecting neurons from DA-induced oxidative damage by efficiently transporting DA into SVs. Studies show that decreased VMAT2 function leads to increased cytosolic DA levels, thereby enhancing vulnerability to neurotoxins and mitochondrial stress [99]. The pathways of DA metabolism are depicted in Fig. (**2**).

The genetic and environmental factors can further disrupt DA metabolism. Polymorphisms in the MAO-B and COMT genes influence enzyme activity and are associated with altered PD risk and progression. Moreover, neurotoxins like 1-methyl-4-phenyl-1,2,3,6-tetrahydropyridine (MPTP) are metabolized by MAO-B into MPP^+^, a potent mitochondrial inhibitor selectively lethal to DA neurons [32, 100]. Understanding DA metabolism is vital not only for elucidating disease mechanisms but also for optimizing therapeutic interventions. The MAO-B inhibitors (*e.g*., selegiline, rasagiline) are used clinically to enhance DA availability and reduce oxidative byproducts. However, these treatments must be carefully balanced to avoid side effects and oxidative overload [101].

The auto-oxidation of DA may result in highly reactive DA quinones (DAQ) and reactive oxygen species (ROS), particularly under basic circumstances [102]. Transition metal ions, including iron, copper, and manganese, or enzyme-like tyrosinase, might trigger DA oxidation. During oxidation of DA, DA-o-quinone (DOQ), a highly reactive, undetectable substance with a brief life span, emerges when two protons and electrons from the hydroxyl groups of DA undergo desquamation. Ambient reductants can convert DOQ back to DA, or the internal cyclization of DOQ in the presence of insufficient ambient reductive forces can further oxidize it to generate reactive aminochrome (AM), a variant of cyclized DAQ [103]. AM is steadier than DOQ and may be recognized, monitored, and described more accurately. DOQ and AM can interact with and conjugate to a range of biomolecules, notably those containing protein residues with cysteine and tyrosine, which have sulfhydryl and hydroxyl groups, respectively. At the same time, AM can form neuromelanin (NM), an insoluble, fragmented pigment in SN [103, 104]. Since NM immediately binds and inactivates radical species under normal conditions, it is regarded as an antioxidant and has been shown to counteract the neurotoxicity of DAQs. Surprisingly, under instances of oxidative stress, NM generates free radicals [105]. The context-dependent redox activity of neuromelanin casts doubt on the notion that it is a universal protective agent. Although NM often quenches free radicals and sequesters redox-active iron, its protective effect depends on extracellular conditions and iron saturation levels [106]. NM transforms into a pro-oxidant state when exposed to high iron concentrations or elevated oxidative stress. Because neuromelanin’s high-affinity binding sites preferentially sequester ferric iron at physiological iron levels, inhibiting the Fenton reaction, this paradox results from its iron-binding kinetics. Nevertheless, the low-affinity sites are occupied when iron concentrations exceed the binding capacity, thereby promoting iron reduction and the production of hydroxyl radicals. Crucially, rather than reducing oxidative tissue damage, incubating human NM with iron increases it [107]. The NM may be implicated in α-syn-associated DA neuronal degeneration. Viral vector-mediated overexpression of VMAT2 dramatically reduced the time-dependent buildup of NM and DA neuron degeneration resulting from tyrosinase overexpression in NM-producing rats that exhibit PD pathology when NM levels exceed a particular pathogenic threshold. Furthermore, reduced NM formation was associated with rats' enhanced motor abilities and DA neuron survival, as well as their diminished emergence of LBs [108, 109].

### 3.1 Protective and Neurotoxic Role of DA in PD

The aminochrome, DOQ, and 5,6-indolequinone are produced before melanin when NM originates *via* oxidation of DA. These compounds exhibit both neurotoxic and neuroprotective effects. VMAT 2 transports DA to the monoaminergic vesicles following its synthesis in the neuronal cytoplasm. This stops the oxidation of DA. Thus, this phenomenon has a neuroprotective function—the oxidation of DA results in DA-o-quinine, which undergoes cyclization and forms aminochrome. The aminochrome molecule undergoes a rearrangement and is changed into 5,6-indolequinone before the synthesis of NM from it. Leu coaminochrome is created by DT-diaphorase when two electrons are reduced in aminochrome. This stops aminochrome from contributing to neurotoxic reactions [110-112]. Moreover, DOQ, aminochrome, and 5,6-indolequinone all exhibit neurotoxic properties, as they form adducts with α-syn and elicit toxicity within the neuron [113]. The α-syn reacts with aminochrome and 5,6-indolequinone to create neurotoxic protofibrils [114]. During the Krebs cycle, oxidative phosphorylation and isocitrate dehydrogenase induce mitochondrial failure [115]. The DJ-1 protein, which is crucial for preventing oxidative stress when mitochondrial electrons are available, is inactivated when DOQ adducts form with α-syn. Any malfunction in the DJ-1 protein contributes to oxidative stress and leads to neurotoxicity [116-118]. Aminochrome's interaction with actin also produces specific adducts. Interaction with neurofilaments is necessary for mitochondria. Nevertheless, the production of these adducts impairs mitochondria's ability to transmit signals to axons and dendrites, leading to neuronal damage. Axonal migration and the integration of vacuoles with lysosomes during autophagy rely upon microtubule growth. Nevertheless, quinines from the adducts with α- and β-tubulin prevent microtubule assembly. As a result, the cell produces toxins, and the autophagy process is disrupted [119, 120]. AM generates adducts and inactivates ubiquitin Carboxy-terminal Hydrolase L1 (UCHL-1). The parkin protein, a ubiquitin ligase of the proteasome, is inactivated by adduct formation, leading to protein and organelle dysfunction. Lysosomal function disruption caused by adduct formation with vacuolar ATPase, which aids the lysosome in maintaining its low pH by generating a H^+^ gradient, contributes to toxicity [121].

## 4 MECHANISMS OF DA RELEASE DEFECTS

### 4.1 SVs Exocytosis

The majority of DA release is mediated by calcium (Ca^2+^)- driven vesicular exocytosis, and its interruption results in a depletion of DA release. Optimal synaptic functionality in DA axons might have been reliant on the establishment of the soluble N-ethylmaleimide-sensitive factor Attachment Protein Receptor (SNARE) complex on the cell membrane by syntaxin-1/SNAP-25 and VAMP-2 (vesicular synaptobrevin-2). SNARE proteins engage exclusively with PD proteins, such as α-syn, alongside LRRK2, and mutations related to PD impair the expression or functionality of the SNARE complex [122-124]. For instance, SNAP25 is inhibited by LRRK2-dependent phosphorylation of snapin, which also reduces the rapidly releasable pool and hinders exocytosis. Furthermore, by binding to the SNARE protein synaptobrevin-2, α-syn oligomers prevent the formation of SNARE complexes [125]. High amounts of mitochondrial ATP are also necessary for exocytosis [126]. Mutations associated with PD and α-syn oligomers can affect mitochondrial transport or function, resulting in synaptic loss and axonal energy depletion [127]. Improper mitochondria could theoretically hinder exocytosis through Ca^2+^ (calcium) disruption, as these cellular structures play a crucial role in Ca^2+^ buffering [128]. Defects in axonal biophysics will result in deficits in DA release, and processes controlling axonal excitability are essential for maintaining the propagation of action potentials. Exocytosis, coming from axons, discharges DA only after the synapse obtains the appropriate impulses [129].

### 4.2 SVs Trafficking and Loading

A shortage of DA in the conveniently dischargeable pool at presynaptic terminals, which may be caused by flaws in its synthesis, vesicular loading, or trafficking, can also impair DA release. DA packing into SVs *via* VMAT2 is closely linked to cytoplasmic DA production. Variations in VMAT2 levels in PD models containing mutations in genes like LRRK2, DJ-1, or VPS35 suggest disturbances in the SV loading mechanism [130, 131]. The vesicular H^+^-ATPase creates the electrochemical gradient between the vesicle and the cytoplasm, which is necessary for the loading of SVs. VMAT2 activity may be impaired by changes in vesicle pH or H+-ATPase activity, which can ultimately lead to a decrease in the quantity of DA released per vesicle [92, 132]. Since mitochondria need ATP to function, they could disrupt this pH gradient. Disrupted synaptic loading causes DA to be lost in the SVs, which not only reduces the amount of DA available for release but also increases cytoplasmic DA, which can be detrimental to the cell since it quickly turns into a reactive species [133, 134]. For a proper DA release, SVs trafficking and/or organization from the pool of SVs to the active site maintains the size of the quickly releasable pool [135].

### 4.3 SVs Pool

The DA release deficiency could also result from direct harm to the SV pool. The production of ROS, a typical PD symptom, may be the root cause of this phenomenon. In addition to providing ATP and regulating Ca^2+^ levels, mitochondria play a crucial role in the formation of synaptic ROS [126, 136]. Increased ROS has been linked to PD models harbouring mutations in DJ-1, Pink1, and SNCA. Moreover, auto-oxidation of cytosolic DA, elevated in PD models, generates more ROS. Numerous causes, including increased dopamine transferase (DAT) activity and defective DA loading into SVs, which are both linked to PD, can result in elevated cytosolic DA [130, 137]. By enabling protons to escape and raising the pH, the reactive DA metabolite DOPAL can harm SVs and encourage the production of α-syn oligomers [138].

### 4.4 Protein Accumulation in the Synapse

One of the main characteristics of PD is protein aggregation. In particular, α-syn aggregation may result in DA discharge defects by physically obstructing cell activities driven by the aggregate or, more accurately, by sequestering the functional protein, thereby disrupting synaptic processes. Defects in DA release occur when oligomeric α-syn and pre-formed fibrils (PFFs) are added without causing genetic changes [139, 140]. Additionally, integration or diminished degradation and elevated α-syn expression are reported in many models of PD-related proteins, including LRRK2, PINK1, and VPS35. This suggests that pathogenic mechanisms could manifest in a number of these models, implicitly *via* α-syn, although to a lesser extent. Nevertheless, α-syn is not the only protein that demonstrates disruptive accumulation and aggregation. According to reports, PD could lead to the protein turnover machinery becoming "clogged," resulting in the buildup of many proteins and a disturbance of synaptic homeostasis [141-143].

### 4.5 SVs Endocytosis, Recycling, and DA Reuptake

Neurotransmitters are replenished in SVs through vesicle recycling and reuptake, which replenishes the vesicular pool for future release events. The DA transporter (DAT) halts neurotransmission and recycles DA for subsequent cycles by acting as an electrogenic Na^+^/Cl^−^ symporter. Through various mechanisms, DAT overexpression and genetic deletion both reduce DA release [144]. Changes in DAT levels have been observed in PD models involving mutations in Vps35, SNCA, LRRK2, parkin, and DJ-1 [130, 131, 145]. Additionally, shortly after exocytosis, tightly controlled endocytosis recovers the SV membrane and associated machinery for subsequent neurotransmission [146]. There is significant indication that SV endocytosis and recycling abnormalities contribute to PD [147]. The phosphorylation of Ras-Associated Binding proteins (RABs), including Rab5b, which are crucial to SV endocytosis and trafficking, is one widely recognized mechanism for LRRK2's involvement in impaired neurotransmission [148]. Moreover, LRRK2 can control endocytosis and SV recycling by phosphorylating auxilin, endophilin A, and synaptojanin1. In PD models, aberrant phosphorylation leads to synaptic abnormalities [149].

### 4.6 Non-cell-autonomous Mechanisms

Axonal dysfunction may not be the only mechanism underlying faulty DA release; other non-neuronal and neuronal networks are also involved in DA release modulation [132]. These neural and non-neuronal striatal networks may have a role in PD defective release, as suggested by studies. In particular, GABA uptake transporters (GATs) on astrocytes in the dorsolateral striatum regulate striatal GABA tone, which tonically inhibits DA release. In a mouse model of PD where human SNCA is overexpressed, these are downregulated, resulting in increased GABAergic inhibition of DA discharge, which suggests impairments in DA release [150].

## 5 PROFOUND PD PATHOGENESIS AND ITS VARIOUS MOLECULAR MECHANISMS

### 5.1 Pathogenic Role of α-syn Aggregation

The concept of PD pathogenesis has been radically transformed, becoming more "synuclein-centric" since the 1997 discovery that certain hereditary forms of PD are caused by point mutations in the α-syn gene, followed by the discovery that LBs are a hallmark of sporadic PD and point variants in the α-syn gene, which are mainly made of misfolded or aggregated α-syn [158]. Genetic evidence linking the SNCA locus to an increased risk of PD has been found in genome-wide association studies (GWAS), suggesting a potential role for α-syn in sporadic PD [159]. The α-syn with 140 amino acids is typically found in abundance in the presynaptic compartment. It is believed to play a role in controlling vesicle dynamics, trafficking, and neurotransmitter release by promoting SNARE complex formation [160, 161]. There are no apparent signs of nerve cell damage or cell death in the striatum of animals with α-syn defects, but there is less DA produced when stimulated. This suggests that the pathogenic role of α-syn in sporadic PD is more likely to involve increased pathological function than loss of function [162]. Previous data indicate that α-syn pathogenicity depends on its aggregation. The buildup of α-syn precedes neuronal death in models of overproduction. Strategies that decrease the aggregative process have been demonstrated to ameliorate motor deficits and neurodegeneration in numerous species, including nonhuman primates, rodents, and invertebrates [163]. Notably, increased α-syn aggregation causes early-onset, rapidly progressive PD, a characteristic of familial PD associated with SNCA mutations [164].

Several internal monitoring systems are in place to ensure protein homeostasis, including the chaperone-mediated process, the ubiquitin-proteasome system, and the autophagy system, which enable the rapid identification and processing or removal of altered proteins [165, 166]. There is mounting evidence that disrupts cellular homeostasis when the aforementioned monitoring systems fail, which in turn causes native and non-native proteins to aggregate (Table **2**) [167, 168]. Mutated versions of α-syn cause the breakdown of surveillance systems, whereas its natural interactions with chaperone proteins, the ubiquitin-proteasome system, and lysosomal proteins reveal its specifics [169-171]. The development of LB inclusions is enhanced by phosphorylated α-syn (at serine 129) [172]. It is also known that α-syn may aggregate due to misfolding [173]. It implies that changes in the natural structure of α-syn may play a role in PD and, therefore, contribute to SN neuron damage. According to reports, the aggregation of α-syn limits the cell's ability to utilize surveillance mechanisms, such as heat shock proteins (Hsp70 and 40), which are trapped along the α-syn in the LB and are hence ineffective [174]. Furthermore, the limitation of chymotrypsin-like 20S/26S protein cleavage and the suppression of RAB1A protein by α-syn aggregation may lead to autophagy deficiencies [175, 176]. Damage to protein homeostasis is a critical component of PD pathogenesis, and this might occur either because monitoring systems fail or because α-syn mediates this failure [177].

### 5.2 Autophagy Dysfunction and Lysosome Pathway

#### 5.2.1 Macroautophagy

Autophagy, also known as macroautophagy, is a self-destructive process in which lysosomes, structures with two membranes, engulf cytosolic cargo (such as aggregates, damaged organelles, or lipid droplets) and then transport them to lysosomes, which have a hydrolytic environment, for breakdown, as shown in Fig. (**3**). Metabolites are then transferred to the cytoplasm, where they are used for energy production or the creation of additional macromolecules [178]. The autophagic flow is severely impaired in PD patients, as evidenced by an increase in autophagosomes and a reduction in lysosomal marker proteins in SN-DA neurons from these patients [179, 180]. Interestingly, autophagy failure has been linked to several PD-related genes. As an example, autophagy is inhibited when α-syn is overexpressed [181]. Furthermore, DJ-1 depletion stimulates oxidative stress-related mitochondrial malfunction [182, 183], which in turn causes microtubule-associated protein 1 to accumulate and serve as an autophagy trigger [184], as shown in Fig. (**3**). In addition, researchers have found that overexpressing the PD-related protein LRRK2 worsens α-syn pathology [185], increases the number of autophagosomes and lysosomal pH, and reduces the activity of hydrolytic enzymes in lysosomes [186].

Research in mice that targets genes *in vivo* confirms that faulty autophagy contributes to the buildup of α-syn. This research demonstrated that presynaptic neuronal accumulation of α-syn occurs when the Atg7 enzyme, necessary for autophagosome formation, is explicitly lost in the brain [187]. Some complexes, including the ubiquitin-binding protein p62 and polyubiquitin, which are linked to K48, were observed in DA neurons following conditional Atg7 deletion, indicating a potential collaboration between the autophagy-lysosomal and proteasomal pathways to alleviate the burden of misfolded proteins [188]. These findings suggest that when one route is selectively impaired, the other may be upregulated to compensate [189]. Moreover, Atg7-deficient mice brains had elevated LRKK2 levels. In line with these findings, they also found that when Atg7 was conditionally deleted from midbrain DA neurons in mice, it caused delayed dementia, early atrophy of dendrites and axons, and prolonged development of motor impairments [187].

#### 5.2.2 Chaperone-mediated Autophagy (CMA)

The pathogenicity in PD is associated with both macroautophagy (also known as autophagy) and CMA. The CMA is a form of selective autophagy that targets a subset of soluble cytosolic proteins bearing a motif recognized by the cytosolic chaperone HSC70 for lysosomal degradation. Post-transcriptionally modified α-syn (*) inhibits CMA, while wild-type α-syn is a substrate for the enzyme. Mutations in LRRK2 and α-syn cause the CMA receptor LAMP-2A to not function as well. However, the mechanism of macroautophagy involves the engulfment of cytosolic components by double-membrane autophagosomes. They are then transported to the hydrolytic environment of lysosomes, where they are degraded. This process destroys unwanted components within cells, including malfunctioning organelles such as mitochondria and defective proteins. In susceptible DA neurons from PD patients, autophagosomes are elevated. To delay neurodegeneration in DA neurons, one may conditionally knock out the autophagy gene Atg7. This gene encodes a protein essential for the formation of autophagosomes. To make up for autophagy deficiencies caused by Atg7 malfunction, UPS proteolysis is enhanced. The autophagosomal membrane component LC3 accumulates in individuals deficient in DJ-1, an inherited PD-related molecule that negatively affects mitochondrial function under oxidative stress. The A53T α-syn mutation promotes cell death and mitophagy. Mutated versions of PINK1 and parkin hinder the elimination of bioenergetically impaired mitochondria, although the proteins typically work together to do this. The lysosomal acidic environment is regulated by the PD-related protein LRRK2, which also influences the number of autophagosomes. The DA toxin MPP^+^ causes the ectopic release of lysosomal proteases following treatment. ROS and the pro-apoptotic protein BAX may mediate this process. People with PD disorders have mutations in genes that encode proteins of the endosomal/lysosomal system: glucocerebrosidase (GBA), type 5 P-type ATPase (ATP13A2), and vacuolar protein sorting-35 (VPS35). To prevent cargo proteins (such as membrane receptors and transporters) from being recycled by lysosomes, VPS35 is an essential component of the retromer protein complex, which regulates retrograde trafficking by binding to the cytoplasmic surface of early endosomes and directing them to the trans-Golgi network. DA neurons lacking VPS35 exhibited abnormalities in α-syn accumulation, late endosome and lysosome enlargement, and LAMP-2A, a CMA receptor, endosome-to-Golgi retrieval. The acidic membrane compartments of lysosomes are home to the ATP13A2 protein. When this enzyme is dysfunctional, lysosomal catabolism is impaired, leading to α-syn accumulation. Glucosylceramide (GC) is degraded in the lysosome by GBA. The risk of PD is higher in those who possess only one mutant GBA allele. Whether or not PD patients have enzyme mutations, their GBA activity is diminished. Oligomeric forms of α-syn (**) hinder GBA's ER-Golgi trafficking and, eventually, its storage in lysosomes, and α-syn acts as a direct inhibitor of GBA bound to the internal surface of lysosomal vesicles, which could explain why GBA is less effective in sporadic PD. Additionally, α-syn soluble oligomers may be stabilized by unprocessed GBA, which accumulates in lysosomes when GBA activity is decreased.

There is evidence that CMA contributes to PD, in addition to macroautophagy [190]. CMA targets the intracellular chaperone HSC70, which identifies certain soluble substances in the cell that have a KFERQ-like domain to direct them for breakdown in the lysosome, as shown in Fig. (**4**). Notably, CMA can bind to wild-type α-syn, which raises the intriguing possibility that PD is associated with decreased CMA activity [191]. Additionally, CMA is inhibited by variants of the PD markers LRRK2 and SNCA that are carcinogenic, as well as α-syn that has been altered post-transcriptionally [192, 193]. Unfortunately, mutant versions of LKKR2 and α-syn proteins cannot be transported to the lysosomal membrane for CMA-mediated destruction, despite being recognized by cytosolic HSC70 and routinely delivered to this compartment [193]. This occurs because these proteins interact abnormally with the LAMP-2A CMA receptor. The fact that PD patients have lower levels of LAMP2A and HSC70 provides further evidence that CMA activity is diminished in PD. Surprisingly, however, individuals with harmful LRRK2 mutations had elevated levels of LAMP-2A, suggesting that they may be attempting to compensate for CMA dysfunction [193, 194].

#### 5.2.3 Lysosomal Enzyme Impairment

The role of the autophagy-lysosome pathway in PD is corroborated by the identification of mutations in genes encoding proteins of the endosomal/lysosomal system, specifically VPS35, a type 5 P-type ATPase, and glucocerebrosidase (GBA). The retromer, of which VPS35 is a component, is a complex of proteins that protects cargo proteins from lysosome-mediated turnover by associating with the cytosolic face of early endosomes and regulating their retrograde transport to the trans-Golgi network [195]. Previous investigation also revealed that patients with autosomal-dominant PD had a mutation in VPS35 [158]. By demonstrating poor endosome-to-Golgi retrieval of the CMA receptor LAMP-2A and larger late endosomes/lysosomes, VPS35-deficient DA neurons in culture corroborated that VPS35 failure significantly affects the autophagic process [196].

It is speculated that ATP13A2, a P-type ATPase, transports cations across the acidic cell membranes of lysosomes [197] (Fig. **4**). Familial Kufor-Rakeb syndrome, caused by mutations in the ATP13A2 gene, is characterized by movement problems and memory loss [198]. Cultured fibroblasts from Kufor-Rakeb syndrome patients were used in experiments that demonstrated the impairment of lysosomal catabolism due to ATP13A2 loss-of-function and the favourability of α-syn accumulation [199]. On the contrary, ATP13A2 co-expression rescues the exaggeration of α-syn in PD models, resulting in the preservation of DA neurons in both animals and cells [197]. Consistent with the idea that the autophagy-lysosome pathway is dysfunctional in sporadic PD, ATP13A2 levels are significantly decreased in the bulk cytoplasm of DA SN neurons from sporadic PD patients and are partially shifted toward LBs. Interestingly, cells with an ATP13A2 mutation also tend to store the mutated protein in the ER, which may lead to ER-stress-induced cell death [200-202]. To break down glucocerebroside (also known as glucosylceramide, GC) into glucose and ceramide, two simpler lipid molecules, the lysosomal enzyme GBA is required. In Gaucher disease, which pathogenic mutations in either the GBA gene may cause, unprocessed GC builds up in several organs, including the brain, spleen, liver, lungs, and bone marrow, due to a severe lack of GBA function [203]. Surprisingly, people who do not get neuronopathic Gaucher disease but do carry a single mutant GBA gene are five times more likely to have PD, suggesting a potential connection between sporadic PD and GBA dysfunction; however, some patients with this type of Gaucher disease also exhibit Parkinsonian symptoms [158, 204].

GBA dysfunction may result from the buildup and aggregation of α-syn, since in the initial stages of PD, GBA protein amounts and function are decreased exclusively in brain areas wherein α-syn levels are substantially elevated [205]. Ultimately, the inappropriate storage of GBA in lysosomes is caused by the oligomeric variants of α-syn, which impede its ER-Golgi trafficking [206]. Additionally, GBA activity inside lysosomal vesicles may be inhibited by α-syn [207]. Experimental data reveal that α-syn misprocessing may be enhanced by reducing GBA lysosomal activity. This is supported by the fact that in a rodent model with Gaucher-related synucleinopathies that are not yet exhibiting symptoms, the formation of α-syn aggregates is reduced, as shown when AAV-mediated expression of GBA was used [208]. Taken together, these findings suggest a positive feedback loop in which neurons undergo neurodegeneration due to an upregulated α-syn, while GBA lysosomal activity is downregulated (Fig. **4**). According to Mazzulli *et al*., one potential explanation for how GBA failure might lead to α-syn-mediated aggregation is that unprocessed GC stabilizes α-syn soluble oligomers [206]. The use of Ambroxol, a secretolytic drug that acts as an activator of GBA function, in PD fibroblasts from a GBA carrier provided further evidence that this approach might be a practical therapeutic method for halting the course of PD [209]. As previously discussed, the buildup and later clustering of α-syn result from malfunctions within the cell's breakdown mechanisms. The ubiquitin-proteasome mechanism and the autophagy-lysosomal cascade may be significantly affected by α-syn assemblies [210].

### 5.3 Synucleinopathy and ER Stress

The ER plays a pivotal role in eukaryotic cell protein folding; disturbances to ER homeostasis may cause misfolded or unfolded proteins to accumulate. Fig. (**5**) shows that when this cellular state, also known as ER stress, is induced temporarily, the body often initiates a natural defensive response called the UPR. This response aims to restore ER homeostasis and normal cell function [211]. However, injured cells are eliminated under persistent ER stress due to the buildup of protein unfolding and ongoing UPR activation, which triggers cell death by activating pro-apoptotic mechanisms. Among the possible triggers of the harmful, long-lasting ER stress response in PD, the accumulation and aggregation of misfolded α-syn are particularly important. Consistent UPR activation indicators have been identified in both vertebrate and mammalian models of synucleinopathy, supporting this concept [212]. Research has shown that α-syn conformers, and more specifically hazardous oligomers, accumulate in the ER during the early stages of illness, along with an increase in Bip (Fig. **5**) [213]. Notably, PD brain tissue has also displayed a similar accumulation of ER-associated α-syn [213]. Supporting this, postmortem research has shown that nigral DA neurons from PD patients experience both ER stress and activation of the under-reporting region [214, 215]. Several studies have also shown a connection between genes related to PD and ER function or alteration, in addition to the chronic ER stress response associated with synucleinopathy. Research on DA neuronal cells dying in *C. elegans* provoked by human α-syn showed that neurons with a mutation in LRRK2 may be more vulnerable to ER stress-driven apoptosis produced by α-syn fold mismatch and aggregation (Table **2**) [216]. A mechanism underlying higher levels of the chaperone Bip, as shown in Fig. (**5**), reduced the impact of LRRK2**.**

Persistent ER stress is a major contributor to neuronal cell death in PD, as previous research suggests; therefore, neuroprotection might be achieved by targeting essential components of the UPR pathway. Several studies have shown that toxic- and genetic-based models of PD may be improved by boosting rather than decreasing UPR components [217-219]. This seemingly paradoxical finding has led to the idea that the resilience of DA neurons could be enhanced throughout PD development through a minor ER stress response, resulting in a persistent, moderate UPR. Neuroprotection in mouse and *Drosophila* PD models was imparted by the ER stress inducer tunicamycin, which induced this sort of response, known as ER hormesis [220]. Therefore, PD treatment options may include fine-tuning of the UPR.

### 5.4 Mitochondrial Dysfunction

It was previously believed that mitochondria served solely as the cell's "powerhouses," but current research suggests that they initiate and transduce signaling pathways involved in many cellular processes, including apoptosis [221]. So, it should come as no surprise that mitochondria play a pivotal role in PD development. Seminal studies on postmortem brain tissue and other PD patient samples, combined with tests in non-human primates and rodents, provide conclusive evidence linking mitochondria to PD development. Neuropathology of PD and intracellular recombinant cell types derived from platelets of PD individuals likewise reveal an impairment in mitochondrial complex I function [158]. In addition, MPP^+^, an inhibitor of mitochondrial complex I, causes selective SN-DA neuronal death in mice and primates when administered systemically as its prodrug, MPTP. The reason for this is that when MPTP was accidentally self-injected into a human vein, it caused permanent degeneration of neurons that appeared and behaved much like those in sporadic PD [158]. These findings are consistent with those of other complex I inhibiting agents, such as the alkaloid rotenone, whose chemical structure is irrelevant to MPP^+^, and the acetogenin annonacin, which, when given systemically to rats, can also cause irreversible damage to the DA-NS pathway. Soursop, a tropical plant, contains the remarkable neurotoxin annonacin, which has been associated with an island of Guadeloupe suffering from an uncommon kind of PD [222]. Since the identification of PD-related genes that govern mitochondrial balance and stress tolerance, researchers have made significant strides in understanding mitochondrial abnormalities in MPS. This discovery adds to the growing body of evidence that in PD, complex I impairment is linked to mitochondrial toxins, which damage the ETC and result in disease severity. Narendra *et al.* (2012) found that the recessive PD-linked genes parkin and PINK1 are involved in mitochondrial dynamics and quality regulation (Fig. **6**) [223]. The proper functioning of the mitochondrial network is essential for cell physiology and survival. Cellular processes that accomplish this goal are known as mitochondrial quality control. Mitophagy, the autophagic removal of malfunctioning or damaged organelles, and mitochondrial biogenesis, the creation of mitochondrial proteins encoded by mitochondrial DNA and nuclear DNA, are all functional components of mitochondrial biogenesis. To repair damaged organelles and degrade them by autophagy, a complex molecular mechanism tightly regulates both fusion and fission. Therefore, to carry out these functions correctly, the mitochondrial network undergoes dynamic evolution, shifting from an extended to a fragmented condition and back again [224]. Last but not least, proper organelle distribution depends on mitochondrial dynamics and transport, particularly at neuronal terminals, where synaptic activity requires substantial energy [225].In addition to their roles in the morphological and functional preservation of the mitochondrial network, the removal of damaged mitochondria, a process known as mitophagy, is regulated by parkin and PINK1 [226, 227]. Unexpectedly, Parkin and PINK1 work together to achieve these goals; PINK1 transports Parkin to damaged mitochondria (meaning they have lost their electrical charge), and then Parkin helps break down these mitochondria through a process known as phagocytosis [228-232].

There is currently a lack of clarity on the exact functional relationship between parkin and PINK1 in defective mitochondria. However, experimental evidence suggests that parkin recruitment and activation may be triggered solely by PINK1 accumulation on the OMM. Fig. (**6**) shows that Parkin regulates the breakdown and elimination of Mfn (1 and 2), which govern mitochondrial anterograde transport; the OMM-associated Miro protein, a Ca^2+^-binding GTPase; and the OMM fusion regulators. In general, it is thought that bioenergetically compromised mitochondria can be more easily removed through the degradation of Mfn1/2 and Miro mediated by the parkin/PINK1 pathway. This is achieved by isolating mitochondria and immobilizing them in the body's organs and tissues, far from the power-hungry ends. Moreover, P62, an adapter protein that binds ubiquitin and LC3, is one mediator of autophagosome engulfment after OMM proteins have been ubiquitinated [233, 234]. In light of these findings, it has been postulated that malfunctioning mitochondria may accumulate and cause mitophagy impairment due to mutations in PINK1 or parkin (230,232) (Figs. **4** and **6**). In contrast to immortalized cell lines, cells derived from human-induced pluripotent stem cells and rat primary neuronal cells were unable to activate mitophagy *via* mitochondrial depolarization when exposed to relatively high concentrations of chemical uncouplers [234, 235]. This has prompted the hypothesis that neurons may have unique bioenergetic needs, which might explain why they are subject to distinct processes regulating mitophagy. However, a recent study shows that, unlike pharmacological uncouplers, the alternative p19ARF translational target smARF can deplete mitochondrial content and enhance mitophagy in neural cells *via* the classic parkin/PINK1-dependent mechanism [236]. Unlike invertebrates, such as flies, the parkin/PINK1 signaling pathway appears to have little control over mitochondrial motility and fusion machinery under normal conditions, but becomes evident only under stress [237, 238]. This disparity is fascinating. Due to their longer life spans and the threat mitochondrial aging poses to cell survival, higher eukaryotes may have evolved mitochondrial quality-control mechanisms that are more strictly regulated. Some research suggests that mitophagy isn't the only mechanism that checks the mitochondria for damage, and redundant systems could play a significant role. In addition to mitophagy, other defense mechanisms include the degradation of unfolded and oxidized soluble proteins by mitochondrial matrix proteases and the rapid formation of mitochondrial-derived vesicles (MDVs), which are directed to lysosomes to remove damaged cargo from mitochondria [239]. Although PINK1, Vps35, and parkin have been implicated in MDV biogenesis, it has not been shown that MDVs play a functional role in PD severity.

Parkin, DJ-1, LRRK2, and PINK1 pathogenic mutations are another cause of mitochondrial dysfunction [240, 241]. As a ubiquitin protease ligase, Parkin encodes E3. Rotenone is an inhibitor of mitochondrial complex I that is very effective in animals that do not have Parkin [241, 242]. A variant of PD, autosomal-recessive PD, is likely caused by mutations in PINK1, which increase α-syn aggregation and decrease mitochondrial respiration and ATP production. It seems that mitophagy and mitochondrial location are both compromised by PINK1 deficiency. Results from studies with *Drosophila* knockouts of both Parkin and PINK1 demonstrated that the two proteins are upstream of each other on the same pathway [243]. The cytosol calls upon Parkin to facilitate the selective autophagic degradation of damaged and depolarized mitochondria. Dysfunctional mitochondria require the accumulation of PINK1 and the activation of kinases for Parkin translocation. Mutations in LRRK2 are linked to autosomal-dominant PD. Abnormalities in mitochondria were seen in the striatum and DA of older homozygous LRRK2G2019S knock-in mice. *Caenorhabditis elegans* neurons with mutated G2019SLRRK2. Typically, dynamin-like protein-mediated LRRK2 mutations are linked to mitochondrial fission, as shown in Fig. (**7**) [41, 244].

Recent research has shown that the nigral nerve cells of PD sufferers have enlarged mitochondria with broken cristae. This data supports the idea that in cases of intermittent PD, mitochondrial quality oversight malfunctions [158]. The mitochondrial complex I inhibitors rotenone and MPTP/ MPP^+^ can reproduce similar morphological changes in mitochondria in experimental settings, suggesting that mitochondrial dynamics may be most impaired in PD due to ETC dysfunction, oxidative stress, and bioenergetic deficits (Fig. **6**) [235]. Multiple publications have demonstrated that α-syn may induce morphological abnormalities, including swelling and disruption of cristae, in addition to interacting with and accumulating in mitochondria [245]. The decreased activity and disintegration of mitochondrial fusion may be addressed by adding PINK1 and parkin when α-syn is overexpressed in the cells and *C. elegans* [246]. This impact may not be caused by blocking the fusion/fission mechanism, but rather by a direct interaction between α-syn polymer chains and the mitochondrial membrane, leading to the membrane's disintegration [247].

The discovery that enhanced mitophagy, mitochondrial loss, and cell death are associated with neural expression of the A53T α-syn mutant further supports the link between disease and mitochondrial quality-control dysfunction. Surprisingly, there is evidence that it is possible to protect neurons against A53T α-syn-induced cellular death and restore mitochondrial function by blocking Drp1 activity, increasing Mfn2 expression, or deleting genes associated with autophagy [248]. Hence, this finding contradicts previous research showing that stimulating macroautophagy and the PINK1/ parkin-associated mitophagy pathway, rather than inhibiting them, has neuroprotective effects in various models of synucleinopathy, such as mice overexpressing wild-type α-syn [181, 249]. Xilouri *et al.* (2009) proposed that the difference between wild-type and mutated A53T α-syn may be due to the former inhibiting the CMA pathway, leading to compensatory overactivation of macroautophagy and, ultimately, the excessive elimination of mitochondria, even those that are not depolarized [250]. The results of this study highlight the need to fine-tune mitophagy for both therapeutic and normal neuronal function, as well as the potential harmful effects of excessive macroautophagy activity. These results also highlight the possibility of techniques targeting disturbed neurons to stimulate mitochondrial elongation. Indeed, filamentous mitochondrial networks provide a more effective response to oxidative stress and other insults by allowing the diffusion of stress molecules throughout the network and triggering compensatory mechanisms [251]. In toxin-based models of PD, neuroprotection provided by the X protein, derived from a virus with anti-apoptotic and mitochondrial targeting capabilities, was linked to mitochondrial filamentation, or the elongation of mitochondria that forms a reticular network [252].

### 5.5 Ca^2+^ Homeostasis and its Dysregulation

The dysregulation of Ca^2+^ homeostasis may substantially influence the selective mortality of DA neurons in PD. While changes in DA neuronal discharge activity are the probable primary source of Ca^2+^ handling abnormalities, α-syn aggregation, mitochondrial dysfunction, and ER impairment are among the several PD-related phenomena that may act as subsequent effects. Abnormal Ca^2+^ levels in the cytosol and subcellular organelles may impact the survival of DA neurons [253, 254]. The DA neurons may undergo Ca^2+^ cyt (cytoplasmic) overload due to hyperactivity of glutamatergic inputs from the subthalamic nucleus, resulting in sustained stimulation of the N-methyl-D-aspartate (NMDA) receptors for glutamate (Table **2**) [41]. Previous research indicates that extracellular glutamate levels are elevated in proximity to SN-DA neurons [255]. The Ca^2+^-dependent pacemaking is another feature of SN-DA neurons. This mechanism operates independently to enhance Ca^2+^ cyt levels through L-type voltage-dependent Ca^2+^ channels, with some of these channels likely featuring a Cav1.3 pore [253]. Due to their poor intrinsic Ca^2+^ buffering capacity, SN-DA neurons may be particularly vulnerable to this type of activity, which helps sustain a baseline DA tone within the striatum [256]. Autonomous pacemaking does indeed enhance the mitochondrial Ca^2+^ burden, which subsequently elevates the onset of mitochondrial oxidative damage in SN neurons in DA [182]. The DA neurons become more susceptible to circumstances with elevated metabolic demand when mitochondrial oxidative stress lowers their bioenergetic reserve capacity [257]. This suggests that isradipine, an L-type Cav channel blocker that inhibits Cav1.3 channels but not Cav1.1 or Cav1.2, reduced baseline mitochondrial oxidative stress in SN DA neurons [182] and provided protection for these neurons against mitochondrial toxins, which are known to impede mitochondrial respiration and energy generation (*e.g*., rotenone and MPP^+^) [253]. Since isradipine did not affect pacemaking, it follows that Ca^2+^ plays a supporting role in regulating DA neuronal activity, but is not necessary for it. Consistent with the gene's proposed function in controlling oxidant defenses, the study found that pacemaker-induced mitochondrial oxidative stress in SN-DA neurons lacking the DJ-1 gene was more pronounced [182].

A novel connection between abnormal proteostasis and impaired mitochondrial function in the pathogenesis of PD has been identified in the perinuclear and dendritic regions of DA neurons, which exhibited intracellular inclusions resembling LBs formed *in vitro* through the recruitment of preformed α-syn fibrils [258]. Consistent with this last finding, a steady rise in brain cells overexpressing the A53T mutant α-syn in mice shows a higher spike rate [259]. This study suggests that L-type Ca^2+^ channel blockers may be beneficial in treating PD. However, epidemiological research has produced conflicting evidence regarding the association between dihydropyridine class L-type Ca^2+^ channel blockers and PD [260]. There are several ways in which excessive Ca^2+^ levels could harm SN-DA neurons. Intracellular damage can occur *via* neurotransmitter autoxidation or post-translational modifications of α-syn, resulting from increased DA synthesis driven by Ca^2+^ elevation, which activates TH [192]. Another way Ca^2+^ excess might harm DA neurons is by activating calpains, a family of cysteine proteases that require Ca^2+^ for their activity. Dufty and their team had demonstrated that LB containing c-terminally calpain-cleaved α-syn was present in the brains of PD patients [261].

Most intriguingly, α-syn-positive aggregates were reduced in human A30P α-syn transgenic mice when the endogenous and selective calpain inhibitor calpastatin was overexpressed [262]. These results are consistent with the knowledge that calpains cleave CDK5, a protein that promotes DA cell death [263]. Therefore, calpains and potential CDK5 inhibitors may represent promising strategies for the treatment of PD. The ability of mitochondria to manage Ca^2+^ *via* uptake or efflux pathways is likely critical for preventing neurodegenerative alterations linked to PD, regardless of the mechanism underlying Ca^2+^ cytotoxicity [264]. This is shown by the fact that mitochondrial Ca^2+^ overload occurs when the PD protein PINK1 is absent, as Ca^2+^ efflux *via* the mitochondrial Na^+^/Ca^2+^ exchanger is reduced [265]. The direct accumulation of Ca^2+^ from the cytosol to the mitochondria *via* the mitochondrial Ca^2+^ uniporter (MCU) may lead to mitochondrial Ca^2+^ overload and subsequent PD-related neurodegenerative processes [182, 266]. The coordinated activation of IP3 receptors on the ER and MCU enables mitochondria, often positioned close to the ER, to take up Ca^2+^ into their matrix [264]. Consequently, the excessive pumping of Ca^2+^ from the cytosol into the ER by the high-affinity sarco-ER Ca^2+^-ATPase could be the cause of Ca^2+^ mitosis and mitochondrial oxidative stress in DA neurons [182, 257]. This process is energy-intensive and could thus exacerbate mitochondrial dysfunction, creating a vicious cycle. The viability of these neurons may also be compromised when pacemaking diminishes, and intracellular Ca^2+^ falls below critical thresholds. However, substantial evidence links stress-induced pacemaking and Ca^2+^ overload to the progression of PD [267]. The following observations really provide credence to this idea: (1) In rodents, the DA toxins rotenone and MPP^+^ reduce firing of DA neurons in midbrain slices at an early stage [268]. To maintain this activity, it is advantageous to inactivate Kir6.2, the pore-forming subunit of K^+^-ATP channels, genetically (2). The neuronal effects of MPP^+^ prodrug MPTP can be mitigated in living organisms by means of the brain-penetrant blocker of small conductance Ca^2+^-activated K^+^ channel apamin [269] and the alkaloid nicotine (NIC) [270]. These compounds increase Ca^2+^ cyt levels in DA neurons and stimulate them electrically [271]. (3) Lastly, Quik *et al.* (2012) found that tobacco smoking lowers PD risk, likely due to the action of its main component, NIC [270]. TH is the rate-limiting enzyme in DA production, and its expression is activity and Ca^2+^ dependent [272]. An intriguing finding was reported by Janezic *et al.* (2013), who observed that aged animals expressing disease-relevant amounts of wild-type α-syn from the full human SNCA locus exhibited lower discharge frequencies in SN DA neurons [273]. Since there is no obvious aggregation pathology in these animals, it is reasonable to conclude that pre-aggregates of α-syn caused diminished spiking. This deficiency might be an early indicator of neurodegeneration. To make sense of these findings, considering evidence linking activity-dependent Ca^2+^ entry to DA cell death, one could hypothesize that DA neurons undergo hypo- and hyperactive phases of degeneration, characterised by Ca^2+^ shortages and excesses, respectively. A mitochondrial genetic model of the MitoPark mouse has shown that quiet DA neurons are physically less affected than hyperactive ones, consistent with this view [274]. The fact that DA neurons may die due to activity and Ca^2+^ shortages suggests that these neurons require Ca^2+^-regulated proteins at a fundamental level. Some cytosolic proteins are essential for life, and PI3K might be one of them [271, 275]. An excellent example of the idea that DA neurons can survive only if Ca^2+^ levels are consistently above a threshold is the way the depolarizing alkaloid nicotine (NIC) protects dying DA neurons in midbrain cultures. To be more specific, the protective effects of NIC have been demonstrated *in vitro* [271] and *in vivo* [276] when Ca^2+^ cyt is elevated above threshold levels by concurrent depolarizing stimuli. This suggests that a7nAChRs, which mediate NIC's protective action, are maintained in an active conformation [276]. Additionally, Ca^2+^ influx was necessary for NIC-mediated neuroprotection under sufficiently depolarizing conditions. DA neurons may be able to control their own survival by drawing on Ca^2+^ stored in the ER. In culture models where neurodegeneration occurs either spontaneously or due to the withdrawal of trophic support or MPP^+^ poisoning, the protection of midbrain DA neurons was achieved by increasing basal cytosolic Ca^2+^ levels *via* activation of ER ryanodine receptor (RyR) channels [277]. The positive effect of ER-Ca^2+^ mobilization was thought to be due to the exertion of Ca^2+^ cyt on a protein target that is essential for DA cell survival. However, it was also suggested that the subsequent reduction in ER-Ca^2+^ load could be advantageous, as it limits mitochondrial Ca^2+^ accumulation resulting from ER-Ca^2+^ mobilisation [257]. Rousseau *et al.* (2013) found that DA neurons were negatively affected by attempts to decrease Ca^2+^ shuttling from the ER to the mitochondria by blocking the ER-IP3R or inhibiting the mitochondrial Ca^2+^ unloading unit (MCU) [278]. This indicates that maintaining Ca^2+^ transfer between the two compartments at a physiological level is crucial, likely to ensure the functionality of the mitochondrial bioenergetic machinery in these neurons and, ultimately, to preserve their survival [279]. There was an early, concurrent decrease in Ca^2+^ and ATP in midbrain cells exposed to the mitochondrial toxin MPP+, consistent with this hypothesis and preceding the death of DA neurons [280]. In addition to reducing Ca^2+^ fluxes into mitochondria, α-syn loss-of-function leads to increased autophagy [282]; therefore, these findings should be viewed in the context of data indicating that the PD protein α-syn facilitates Ca^2+^ mitochondrial transients induced by IP3R activation. In line with these findings, α-syn was shown to bind to mitochondria-associated ER membranes, a structurally and functionally unique subdomain of the ER [281]. The activation of IP3Rs by two other PD-associated proteins, Parkin and DJ-1, has been shown to facilitate ER-mitochondria tethering [280-284]; however, this process is hindered by mutant DJ-1 and Parkin siRNA (small interfering RNA). The potential role of Ca^2+^-related events in DA cell loss in PD is summarized in Fig. (**8**).

The ER mitochondria Ca^2+^ shuttling mechanism is enhanced by wild-type PD proteins, including parkin, α-syn, and DJ-1. Still, it is inhibited by mutant versions of these proteins or by siRNA silencing of parkin. Treatments that bring mitochondrial Ca^2+^ levels back up to appropriate levels provide neuroprotection, whereas low levels of Ca^2+^ may cause DA cell death. The lack of the PD protein DJ-1 and the increase of intracellular α-syn LBs, such as inclusions, amplify mitochondrial oxidative stress induced by pace-making in the hyperactive state. The spike rate in nigral DA neurons is similarly increased by overexpressing A53T mutant α-syn. Ca^2+^ mitosis occurs when the PD protein PINK1 is deficient, which reduces the efflux of Ca^2+^ from mitochondria. The capacity of bioenergetic reserves in DA neurons is diminished, making them more vulnerable to mitochondrial toxins such as MPP^+^ and rotenone due to heightened baseline mitochondrial oxidant stress. Isradipine, an L-type Ca^2+^ channel blocker, exhibits a specific affinity for Cav1.3 channels. By reducing baseline mitochondrial oxidant stress, isradipine enhances the resilience of SN DA neurons against mitochondrial toxins. The other possibility is that the increased Ca^2+^ cyt becomes harmful to SN-DA neurons because it activates TH, which in turn stimulates DA production. This leads to intracellular damage *via* neurotransmitter autoxidation (*) and post-translational changes of α-syn (**). The activation of calpains, which are Ca^2+^-activated proteases, is another mechanism by which Ca^2+^ excess may have harmful consequences. LBs indeed included α-syn fragments split by calpain. Keep in mind that NMDA glutamate receptors are involved in the process by which subthalamic nucleus (STN)-mediated excitotoxicity might worsen aberrant Ca^2+^ influx.

## 6 MOLECULAR TRIGGERS AND GENE SUSCEPTIBILITY IN PD

Neuronal homeostasis is disrupted, and the risk of disease onset is elevated by both heritable mutations and environmental exposures, which are encompassed within the molecular etiology of PD [282].

### 6.1 Genetic Mutations

Familial and sporadic PD have been causally linked to mutations in a subset of genes. The α-syn, encoded by the SNCA gene, is aggregated in a manner that constitutes a pathological hallmark of PD [283]. Its misfolding and aggregation are promoted by mutations or duplications in SNC4. The PINK1 and PARK2 (Parkin) are required for mitochondrial quality control through mitophagy; their absence causes poor mitochondrial evacuation and increased oxidative stress [284]. LRRK2 mutations, particularly G2019S, are the leading cause of autosomal dominant PD. Increased kinase activity has been linked to mutations that cause abnormal protein phosphorylation and neuroinflammation [285]. DJ-1 supports the oxidative stress response, and its deficiency compromises cellular ROS defense mechanisms [286].

### 6.2 Environmental Toxins

Exposure to environmental neurotoxins has been implicated in sporadic PD cases. DA neurons are selectively targeted by compounds such as MPTP, rotenone, and paraquat. MPTP is metabolized to MPP^+^, by which mitochondrial complex I is inhibited, resulting in ATP depletion and ROS generation [287]. Similarly, rotenone, a widely used pesticide in the development of PD models, causes mitochondrial damage. Paraquat causes oxidative stress through redox cycling and the formation of superoxide radicals, which contribute to the death of DA neurons. These environmental assaults replicate the molecular hallmarks of PD, providing insights into disease mechanisms [288].

### 6.3 PD translational Models

Animal and cellular models have been proposed as beneficial for studying PD and testing potential therapies. Toxin-based models, such as MPTP and 6-OHDA, cause DA neuron degeneration by inducing oxidative stress or mitochondrial damage (Table **3**) [289]. 6-OHDA causes localised, rapid disruption to the NS pathway in rats; MPTP is routinely employed in primates and mice (Table **3**). Genetic research helps shed insight into familial PD. Overexpression of the α-syn gene (SNCA) in transgenic animals mimics the protein aggregation associated with PD [290]. Gene knockout models, such as those for Parkin and LRRK2, are used to explore autophagy issues and mitochondrial dysfunction. Models established from induced pluripotent stem cells (iPSCs), while resource-intensive and requiring advanced procedures, enable human-based cellular systems to examine patient-specific genetic backgrounds and issues [291].

### 6.4 Protective Factors

Caffeine and nicotine affect the pathophysiology of PD through complementary neuroprotective mechanisms. Adenosine A2A receptor antagonism is the primary mechanism by which caffeine, the most widely used psychoactive stimulant, provides protection. Mechanistically, caffeine reduces neuroinflammation by reducing astrogliosis and microglial activation, increases autophagy-dependent α-syn degradation *via* chaperone-mediated autophagy and macroautophagy pathways, and changes the composition of the gut microbiota to promote anti-inflammatory traits [297, 298]. Nicotine operates through distinct nicotinic acetylcholine receptor (nAChR) subtypes, predominantly α4β2* and α6β2*, providing neuroprotection against DA neurotoxins. Nicotine increases the expression of trophic factors, including brain-derived neurotrophic factor and fibroblast growth factor-2, and reduces L-DOPA-induced dyskinesias by 35-50% in non-human primates [297, 299].

## 7 MECHANISMS OF NEURODEGENERATION AND PD

Multiple cell death pathways are involved in the mediation of neuronal death in PD, with each contributing uniquely to the progressive degeneration of DA neurons [300].

### 7.1 Apoptosis and Caspase Activity

Apoptotic pathways are common in PD, including intrinsic (mitochondria-dependent) and extrinsic (death receptor-mediated) mechanisms. The release of cytochrome c from mitochondria activates caspase-9, which activates caspase-3, the primary apoptosis executioner (Table **4**) [301-303]. This results in DNA fragmentation and cell disintegration. The Pro-apoptotic (Bax, Bak) and anti-apoptotic (Bcl-2) proteins control mitochondrial outer membrane permeabilization (MOMP) [304].

### 7.2 Autophagic Cell Death

Although autophagy is normally cytoprotective by removing damaged proteins and organelles, dysregulation can cause autophagic cell death. In PD, excessive or defective autophagic activity causes neuronal self-digestion, particularly when lysosomal degradation is compromised [305]. The accumulation of autophagosomes and failure to complete autophagic flux aggravate cellular stress and damage [306].

### 7.3 Ferroptosis

Ferroptosis is a type of non-apoptotic cell death that is distinguished by the formation of lipid peroxides in the presence of iron (Table **4**) [307, 308]. It functions differently from apoptosis and necrosis [309]. In PD, higher iron levels in the SN and decreased glutathione peroxidase 4 (GPX4) activity enhance lipid ROS production, leading to membrane damage (Table **4**). Ferroptosis is defined by the inhibition of GPX4 and the overexpression of acyl-CoA synthetase long-chain family member 4 (ACSL4) (Table **4**) [310].

## 8 NEUROPROTECTIVE STRATEGIES IN PD

The pathological processes contributing to DA neuron death are being counteracted through the investigation of several neuroprotective strategies [314].

### 8.1 Antioxidant Therapies

Reactive oxygen species are targeted for neutralization by antioxidants such as Coenzyme Q10, Vitamin E, and N-acetylcysteine (NAC), and mitochondrial integrity is maintained (Table **5**). The redox buffering capacity is enhanced, and oxidative damage is mitigated by these compounds, particularly within the mitochondria-rich environment of the SN [315].

### 8.2 Mitochondrial Protection Agents

Creatine and MitoQ are molecules that target mitochondrial dysfunction by increasing ATP synthesis, stabilising mitochondrial membrane potential, and decreasing ROS generation (Table **5**) [316]. Preclinical studies have demonstrated that MitoQ, a mitochondria-targeted antioxidant, can preserve mitochondrial morphology and prevent DA neuronal death.

### 8.3 Anti-inflammatory Compounds

The chronic neuroinflammation contributes to the course of PD, and anti-inflammatory drugs seek to block this process. NSAIDs, minocycline, and TNF-α inhibitors can reduce cytokine production and microglial activation, resulting in neuroprotective effects in animals (Table **5**) [317-320].

### 8.4 Proteostasis and Autophagy Modulation

Another treatment option is to stimulate protein degradation mechanisms such as autophagy and the ubiquitin-proteasome system. Rapamycin, a mTOR inhibitor, promotes autophagy, which aids in the clearance of misfolded proteins, such as α-syn. Trehalose and other proteostasis enhancers promote lysosomal breakdown and cell homeostasis [321].

## 9 EMERGING GENE AND CELL THERAPIES

### 9.1 Gene Therapy Approaches

Gene therapy for PD aims at restoring DA synthesis or providing neurotrophic support employing viral vectors [327, 328]. Adeno-associated virus (AAV)-mediated AADC gene transfer improves DA synthesis by converting levodopa more effectively. Similarly, the use of neurotrophic factors such as glial cell line-derived neurotrophic factor (GDNF) serves to protect and restore DA neurons (Table **6**) [329]. Such therapies are currently in early to mid-stage clinical investigations. Gene therapy for PD aims to restore DA synthesis or provide neurotrophic support using viral vectors.

AAV vectors targeting basal ganglia structures have been used in 9 gene therapy clinical trials that have demonstrated remarkable safety, with no significant adverse events related to gene therapy reported in any of the trials. None, however, attained strong enough clinical efficacy to be approved by regulators, demonstrating a fundamental mismatch between preclinical animal models and human outcomes [300].

### 9.2 Stem Cell-based Regeneration

Clinical trials are being carried out in humans to evaluate safety, graft survival, and functional recovery. The goal of stem cell therapy is to replace missing DA neurons [330]. iPSCs are transformed into DA neurones in the midbrain before being transplanted into the striatum (Table **6**) [331]. Tissue stem cell approaches demonstrate improved safety and efficacy trajectories. The Phase 1 bemdaneprocel trial (human embryonic stem cell-derived dopaminergic progenitors) demonstrated sustained engraftment after discontinuation of immunosuppression, with no serious cell therapy-related adverse events, and encouraging secondary motor improvements at 18 months. Standardized methods are lacking in 33 assessed research studies, which have varied designs (most are preclinical). Although autologous transplants have shown better safety and efficacy than allogeneic methods, there is still a lack of long-term follow-up data and insufficient information on the tumorigenicity risks associated with therapies derived from embryonic stem cells in human populations. Phase 3 effectiveness data are still pending [332].

### 9.3 CRISPR and RNA-based Therapies

Genome editing techniques like CRISPR-Cas9 can fix harmful mutations in genes like SNCA and LRRK2. Antisense oligonucleotides (ASOs) can suppress the production of harmful gene variants or alter splicing (Table **6**) [333]. These RNA-based medicines are highly selective and are being tested in preclinical animals, with encouraging results.

## 10 MANAGEMENT APPROACHES FOR PD

PD can be treated with surgery, medication, and lifestyle changes (Table **7**). Pharmacologically, Levodopa is the most effective treatment because it acts as a DA precursor [337, 338]. It is often combined with carbidopa for better CNS availability. Alternatives include DA agonists and MAO-B inhibitors, both of which have their own adverse effects. In advanced PD patients, surgical treatments such as deep-brain stimulation (DBS) are used, and electrical regulation of basal ganglia circuits results in significant alleviation of movement symptoms (Table **7**) [339]. However, thorough patient selection and postoperative monitoring are considered crucial. Non-pharmacological treatments, such as physical therapy and exercise, are considered essential for maintaining motor and cognitive function (Table **7**). Emerging treatments, including gene therapy and cell transplantation, attempt to provide neuroprotective or regenerative effects; however, they are still in the experimental phase [340].

## 11 FUTURE DIRECTIONS AND CONCLUSIONS

Many factors contribute to the development of PD, a neurological ailment for which a cause is still mostly unknown. The predominant clinical hallmark of the illness, a progressive impairment of voluntary motor control, is caused by a loss of DA neurons in the SN. Cell-autonomous pathways that contribute to DA cell death in familial and sporadic variants of the illness. Specifically, DA neurons could arise from cellular disturbances linked to protein misfolding and aggregation, disruptions in autophagic catabolism, ER stress, mitochondrial dysfunction, or a lack of Ca^2+^ homeostasis, all of which can cause neuronal death. We have made significant progress in understanding the pathophysiology of PD in just 20 years. Before the discovery of α-syn, the predominant focus of investigations into the pathomechanisms of PD had been on mitochondrial bioenergetic deficiencies, oxidative damage, and cell death *via* apoptotic pathways. Modern breakthroughs in molecular research have enabled the development of methods that target specific disease-causing processes, such as the clumping of α-syn, mitochondrial dysfunction, and genetic abnormalities like LRRK2 and GBA1. At the same time, DA neurons derived from pluripotent stem cells have become an ethically acceptable and scalable option for cell-replacement therapy. The safety and functional integration of these grafts have been shown in early-phase clinical studies. Improvements in immunological compatibility, anatomical accuracy, homotopic transplantation, and circuit-level reconstruction are now the subjects of active research. These new approaches can change the way we think about PD therapy by addressing both its symptoms and its underlying causes.

The dissemination of α-syn disease is probably aided by non-cell-autonomous pathways as well. Increasingly, research suggests that α-syn may accumulate in newly "contaminated" neurons after undergoing a harmful templated conformational shift. This scenario indicates that seeding-prone exogenous α-syn aggregates may not be cell-autonomous and may contribute to disease development. Although there is substantial experimental evidence for α-syn spreading, there is currently no proof that it occurs in a pathophysiological setting at levels of α-syn relevant to disease. As our understanding of PD continues to expand and more data are collected through advances in high-throughput multi-omics profiling, it is expected that increasingly comprehensive approaches will be necessary. These methodologies will be instrumental in identifying novel therapeutic targets, predicting the impact of treatment on the disease, and informing treatment strategies. In alignment with the second point, it is increasingly evident that various forms of PD can emerge from different causes; nonetheless, they all exhibit the pathological hallmark of a significant and preferential loss of nigral DA neurons and, to a lesser extent, synucleinopathy. Approximately 10% of PD cases are attributable solely to environmental or genetic factors, the two primary etiological categories. In contrast, spontaneous PD often occurs in individuals with loss-of-function mutations in GBA, a genetic abnormality that affects crucial cellular mechanisms involved in brain homeostasis.

## Figures and Tables

**Fig. (1):**
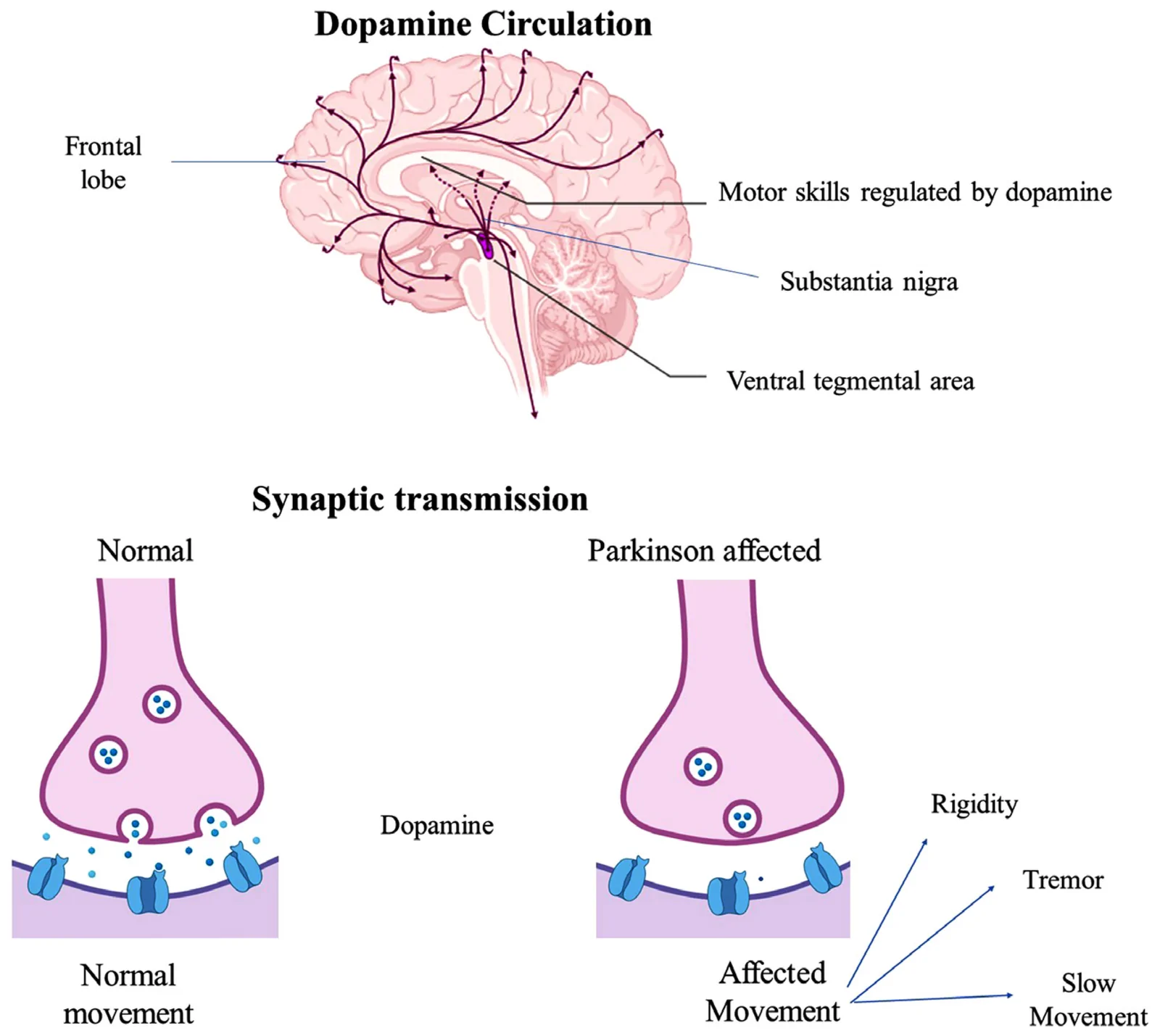
Illustrating the degeneration of DA neurons in the SN region, which is the primary reason for the slowness, stiffness, impaired motor function, and tremors experienced by PD patients.

**Fig. (2):**
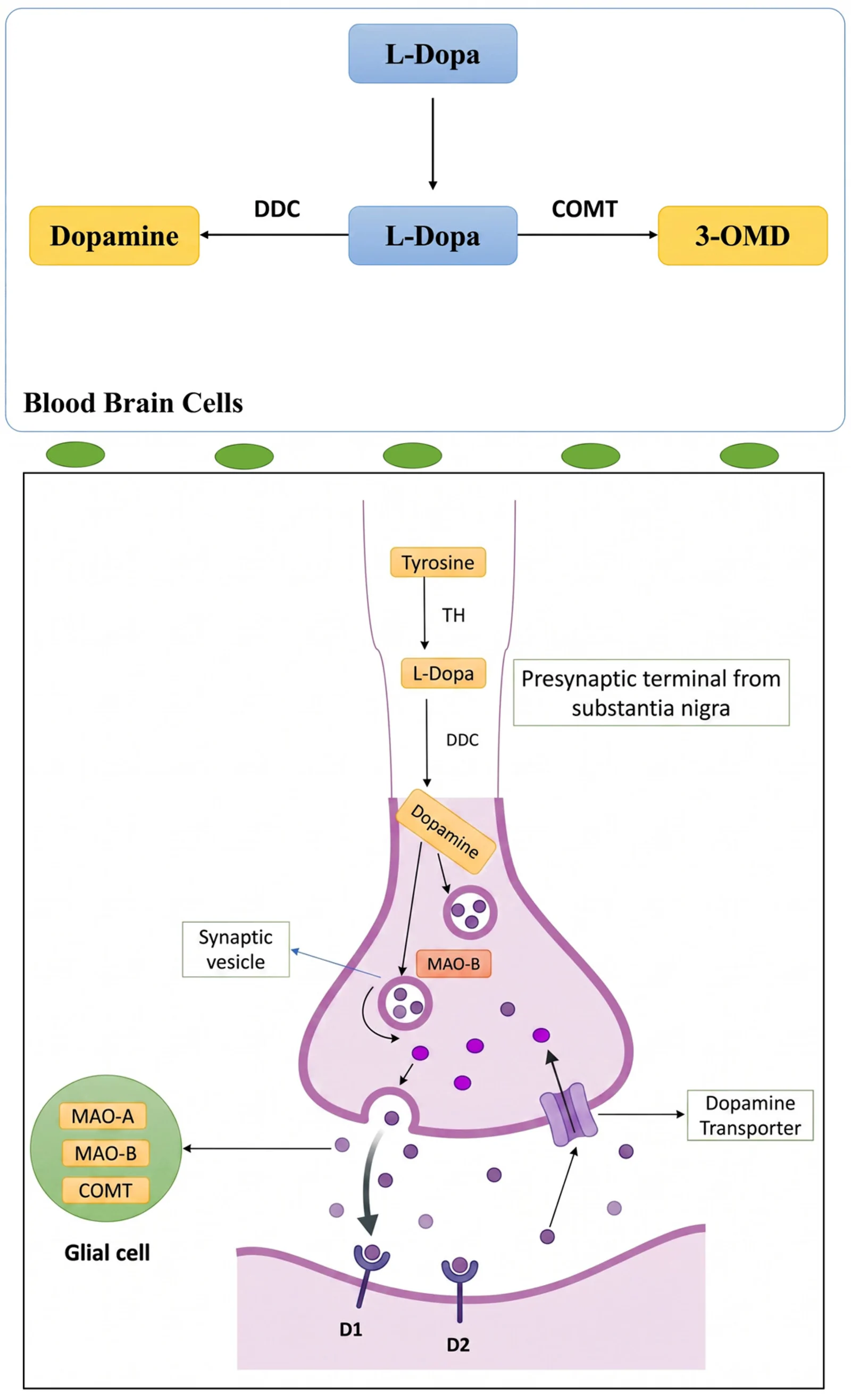
Indicates the schematic illustration of metabolic pathways for DA. L-DOPA is converted to dopamine by DOPA decarboxylase (DDC), producing 3-O-methyldopa (3-OMD), which is further metabolised by COMT, which crosses the BBB. The SN's presynaptic DA neurotransmission is shown in the bottom half of the figure. The DA is also synthesized from tyrosine *via* the enzyme TH and released into the synaptic cleft to activate D1 and D2 receptors. Its activity is then regulated by reuptake *via* the DA transporter and breakdown by COMT and MAO (A/B).

**Fig. (3):**
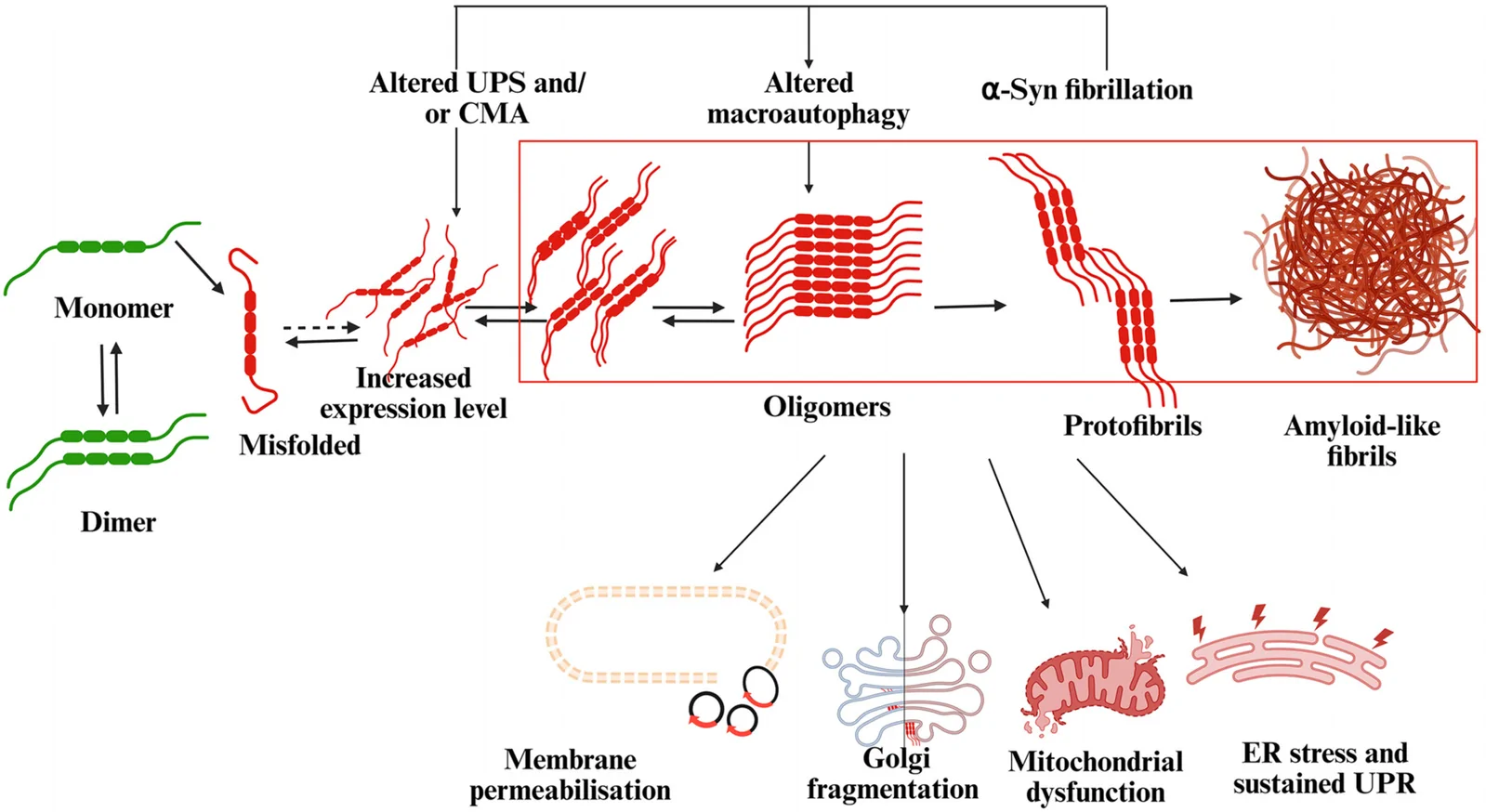
Potential causes and effects of α-syn aggregation in PD. Neuronal cell death in PD is primarily caused by α-syn misfolding and aggregation, as evidenced by compelling and ever-increasing data. During α-syn fibrillation, unfolded or misfolded monomers aggregate into dimers, which subsequently form small oligomers that interact with β-sheets, potentially stabilizing them. The progression from α-syn aggregation to insoluble protofibrils with a larger molecular weight, and finally to amyloid-like fibrils that may aggregate into pathogenic formations, such as LBs, occurs at this stage. An imbalance between expression and degradation processes might lead to oligomer formation by gradually accumulating full-length wild-type α-syn, which is one of the factors that initiate or favour α-syn fibrillation. For instance, the buildup of misfolded α-syn that promotes future fibrillation may be caused by a malfunction of the ubiquitin-proteasome system (UPS) and a breakdown of the chaperone-mediated autophagy (CMA) process. The poor removal of oligomers and fibrils, which have a higher molecular weight than α-syn, by macroautophagy may also be due to impairments in the autophagy-lysosome pathway. It is believed that α-syn oligomers and protofibrils are particularly detrimental to neurons. Among their many potential effects on cells are alterations in mitochondrial and endoplasmic reticulum (ER) homeostasis, increased fragmentation of the Golgi apparatus, and membrane permeabilization. The inspiration and acknowledgement for the creation of this illustration was recreated using www.Biorender.com, as shown in this article [158].

**Fig. (4):**
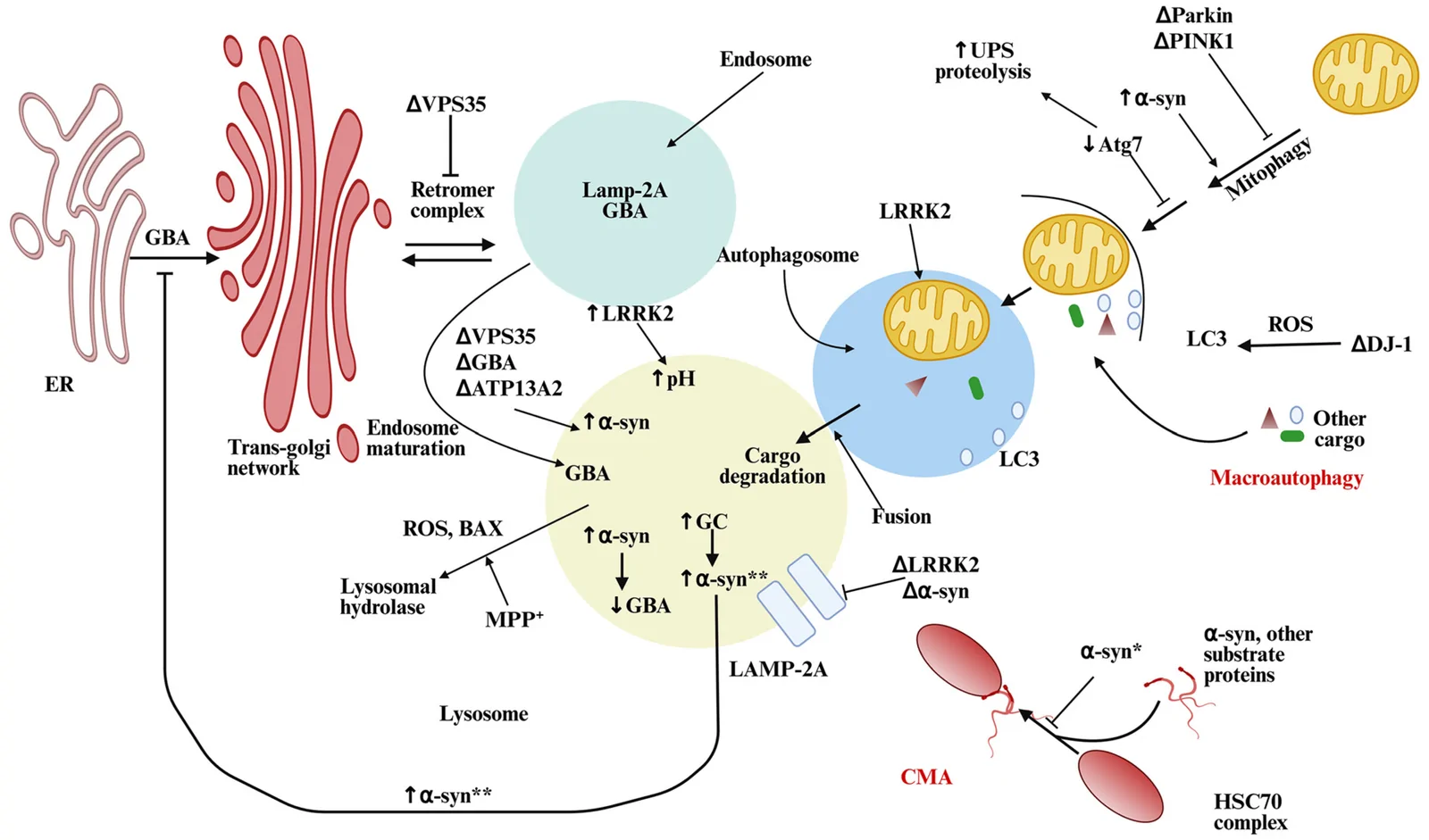
Autophagy-lysosome system dysfunction in PD. The illustration's description is provided in the chapter on chaperone-mediated autophagy. The inspiration and acknowledgement for the creation of this illustration was recreated using www.Biorender.com, as shown in this article [158].

**Fig. (5):**
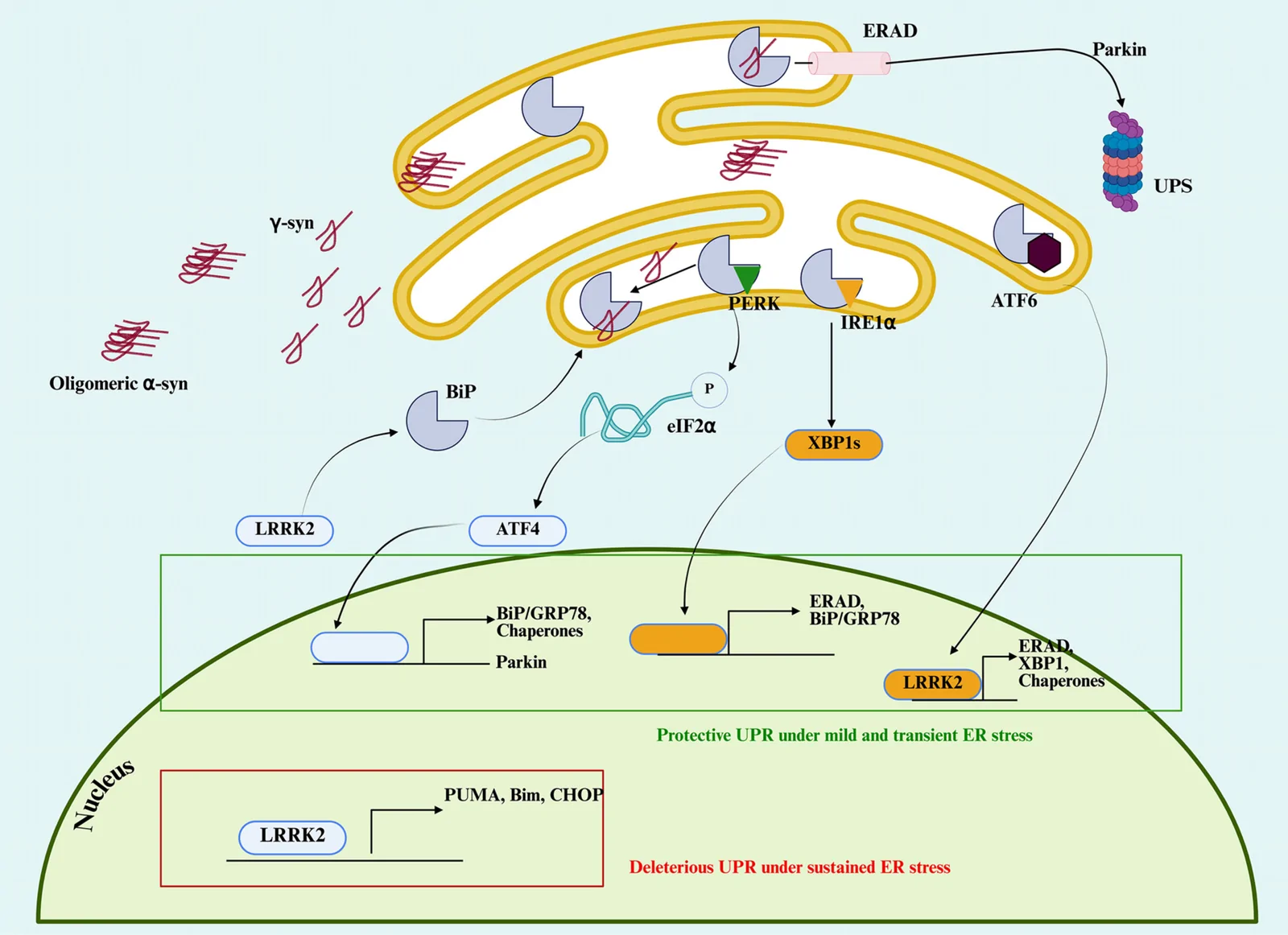
ER stress and protein misfolding responses in PD. Misfolded or unfolded α-syn may accumulate and clump together in PD, leading to ER stress and the activation of death processes. In response to ER stress, a complex molecular network known as the unfolded protein response (UPR) is typically activated. This network includes stress sensors located at the ER membrane (IRE1a, PERK, and ATF6), which, through various mechanisms and intermediate factors, transmit an unfolded protein signal to the nucleus. Importantly, IRE1a promotes X-box binding protein 1 (XBP1) splicing, abbreviated as XBP1s, and the activation of PERK leads to the phosphorylation of eIF2α, which in turn stimulates the activation of activating transcription factor 4 (ATF4). Once in the nucleus, XBP1s and ATF (4 and 6) control gene transcription by binding to certain DNA-responsive regions. Upon binding to the luminal domains of the stress sensors, Bip, a member of the 70 kDa family of heat shock proteins, acts as an ER chaperone, keeping the stress sensors inactive. However, upon binding to unfolded α-syn, Bip dissociates from the sensors and activates them. Subsequently, the UPS and ER-associated degradation (ERAD) pathways aim to break down Bip-α-syn complexes. Notably, Parkin, a component of the UPS and an E3 ubiquitin ligase, is increased in response to Unfolded protein breakdown *via* the ERAD pathway, which may be facilitated by ER stress. Also, by promoting Bip expression, LRRK2 may contribute to the protective UPR. At the nuclear level, UPR-mediated gene regulation results in the breakdown of unfolded proteins *via* the ERAD system, the activation of ER chaperones, and a reduction in protein synthesis. However, unfolded proteins accumulate, and continuous UPR activity activates pro-apoptotic pathways, leading to cell death in response to chronic ER stress and the elimination of damaged cells. Specifically, ATF4, a transcription factor that modulates several transcriptional factors, is a downstream regulator of the PERK/eIF2α network, which induces apoptosis. This network includes the C/EBP homologous protein (CHOP) and pro-apoptotic proteins PUMA and Bim. The inspiration and acknowledgement for the creation of this illustration were sourced from www.Biorender.com, as shown in this article [158].

**Fig. (6):**
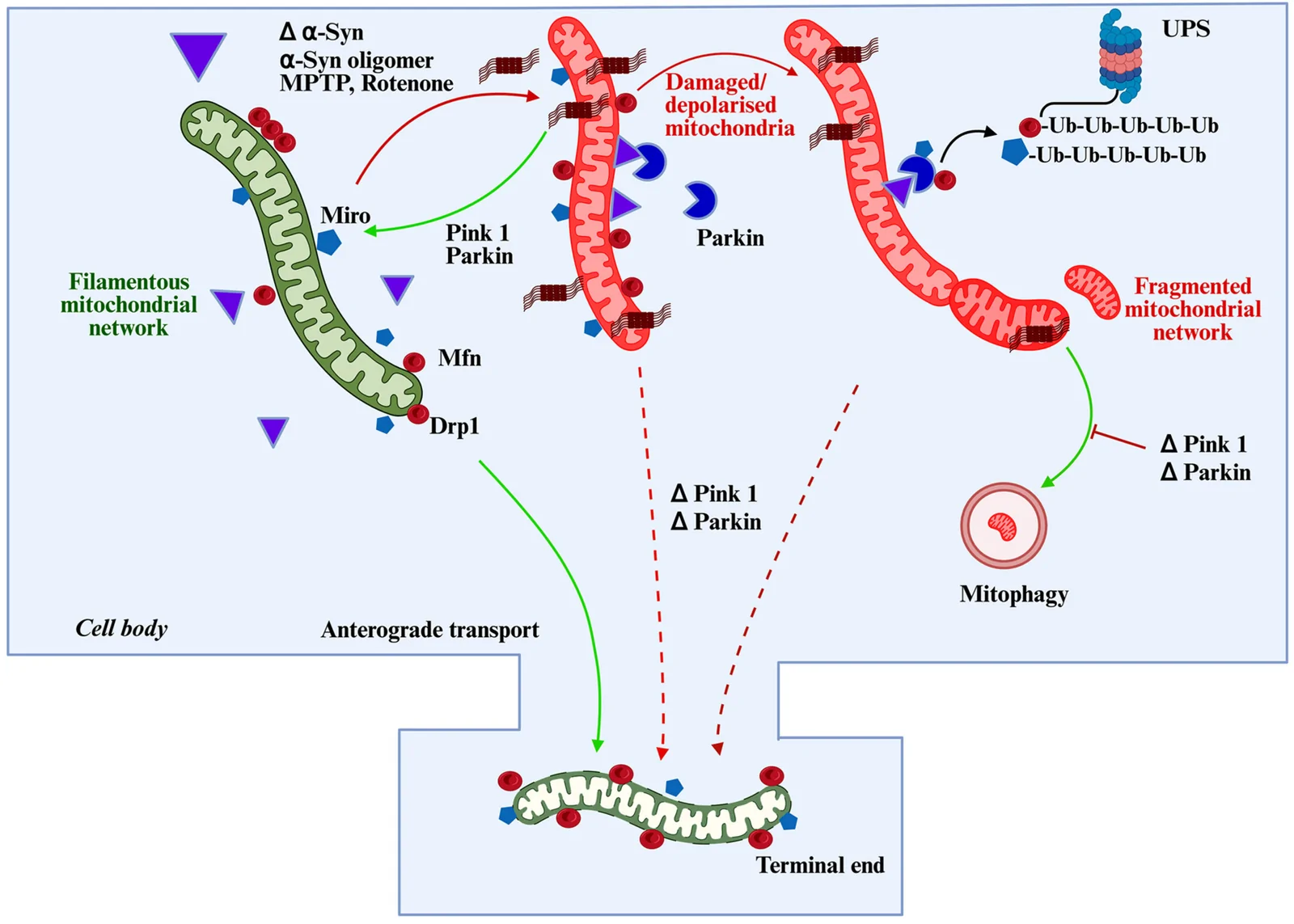
PD and mitochondrial dysfunction: Accumulation of α-syn oligomers causes mitochondria to fragment at the molecular level. The mitochondrial toxins rotenone and MPTP, which are associated with PD and oxidative stress more generally, may cause mitochondrial networks to fragment, a characteristic of malfunctioning mitochondria. The involvement of Parkin and PINK1 in mitophagy, the process by which damaged mitochondria are broken down and removed, is strongly supported by the available data. The breakdown mechanism involving the ubiquitin-proteasome system (UPS) is activated when damaged or depolarized mitochondria attract and accumulate PINK1 at the outer mitochondrial membrane (OMM). The Parkin gene is recruited and activated by PINK1. The OMM fusion-prone protein mitofusin (Mfn) is ubiquitinated in a Parkin-dependent way, which causes the UPS to damage it. Then, the compromised mitochondrial circuit may be more readily fragmented by Drp1, a protein that controls the final stage of mitochondrial fission. Mitophagy is the process by which damaged mitochondria are engulfed by autophagosomes and delivered to lysosomes for degradation. In addition to Mfn, parkin, and UPS, are required for the degradation of Miro, another crucial OMM protein that plays a role in the axon-dependent anterograde transport of mitochondria. Degradation of mitochondria slows the transport of damaged mitochondria to their ends, thereby facilitating the removal of fragmented organelles through mitophagy. Mitophagy is impaired, and abnormal mitochondria accumulate when PINK1 and parkin abnormalities disrupt mitochondrial quality control and dynamics. The illustration was recreated using Biorender, as described in [158].

**Fig. (7):**
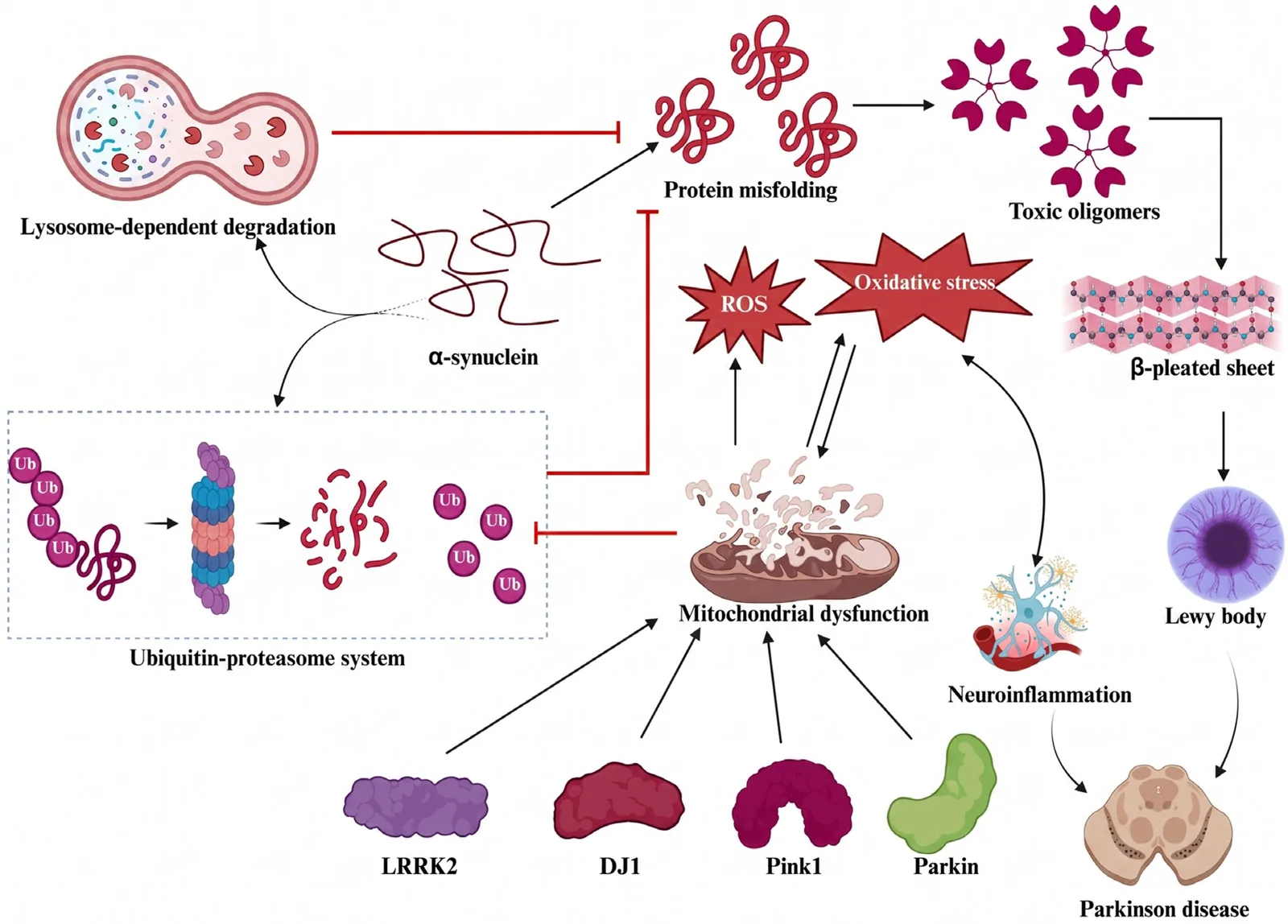
The ubiquitin-proteasome and lysosomal autophagy processes maintain stable intracellular α-syn levels. The buildup of α-syn might be caused by mitochondrial dysfunction, neuroinflammation, or oxidative stress that hinders these degradation processes. In addition, mitochondrial dysfunction and increased cell death are caused by mutations in genes such as LRRK2, DJ-1, Parkin, and Pink1. The inspiration and acknowledgement for the creation of this illustration were sourced from www.Biorender.com, as shown in this article [41].

**Fig. (8):**
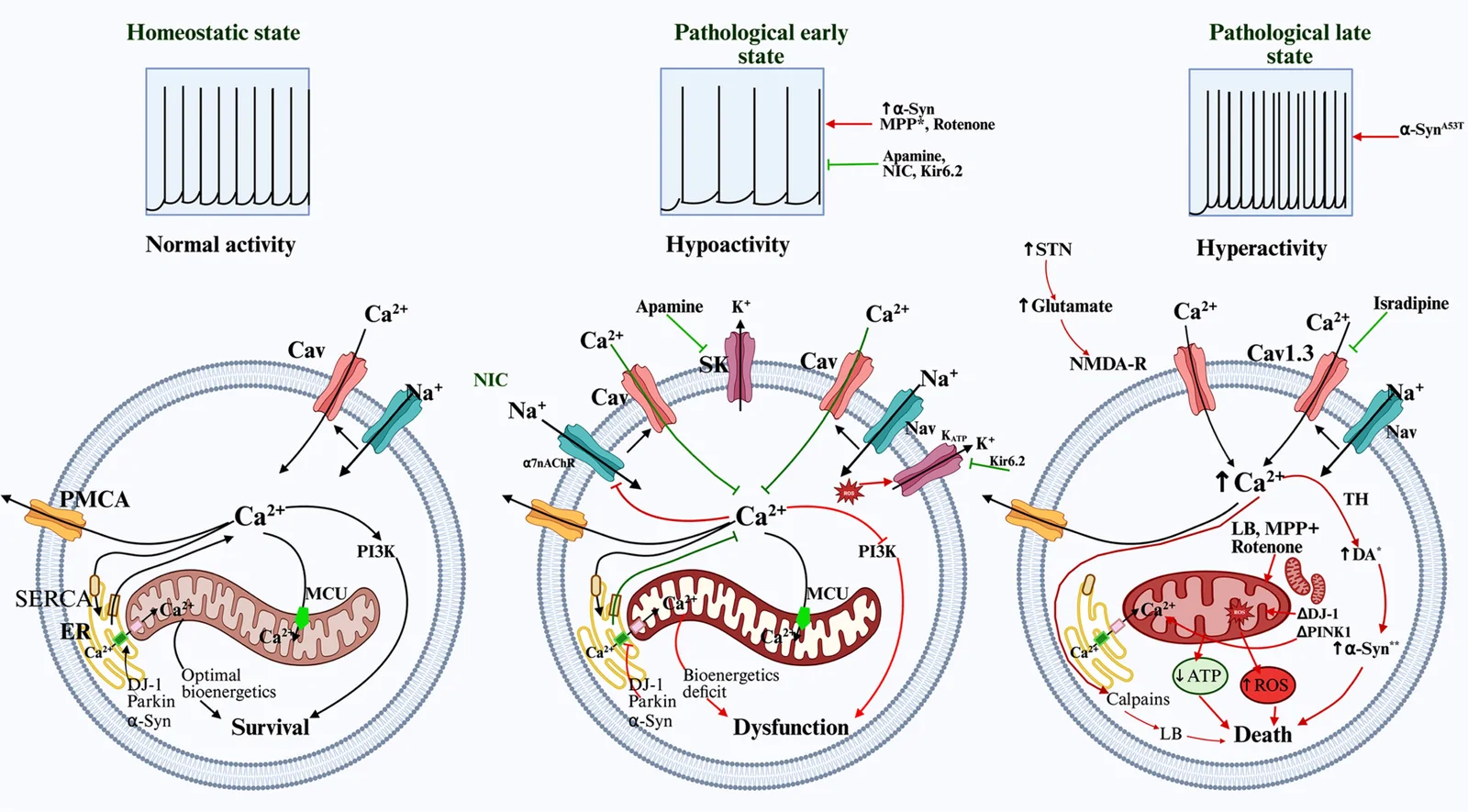
The function of activity-and Ca^2+^-dependent processes in PD-related DA cell degeneration. Changes in DA neurons' Ca^2+^ handling and discharge patterns make them more susceptible to these changes. Degeneration causes hypo- and hyperactive states when Ca^2+^ is in short supply and in excess, respectively. When they are in their homeostatic state, DA neurons utilize a combination of Na+ channels (Nav) and L-type voltage-dependent Ca^2+^ channels, some of which carry the Cav1.3 pore, to drive autonomous pacemaking. Primarily, pacing raises cyt Ca^2+^ and, secondly, mitochondrial Ca^2+^. The MCU is involved in the direct or indirect transfer of Ca^2+^ from the cytosol to the mitochondria. In the second scenario, the high-affinity sarco-ER Ca^2+^-ATPase (SERCA) pumps Ca^2+^ from the cytosol into the ER, where it is then transported to the mitochondria by the sequential activation of the ER-IP3R and MCU. Raising mitochondrial Ca^2+^ levels triggers baseline mitochondrial oxidative stress in SN-DA neurons, which, in turn, increases their ATP requirement to maintain correct cytosolic Ca^2+^ concentrations *via* SERCA and the PMCA. As their activity decreases and the baseline Ca^2+^ cyt level falls below threshold values, DA neurons become more susceptible to the hypoactive state. These alterations may come first in the degeneration process, just before a period of hyperactivity. Mitochondrial toxins, such as rotenone and MPP^+^, as well as disease-relevant levels of α-syn expression, can be used in experiments to reduce DA neuronal activity. A genetic blockage of KATP channels, such as those that are activated in response to oxidative stress, or the stimulation of neurons that produce DA by the alkaloid nicotine (NIC) or apamine, a component of the venom of bees that may protect against PD toxins. To defend itself, apamine blocks SK channels, whereas NIC activates nAChRs. By keeping Ca^2+^ cyt above threshold levels (but below toxic levels), NIC (nicotine) protects and activates survival-critical protein targets, such as PI3K. Because optimal mitochondrial bioenergetics requires Ca^2+^ shuttling from the ER to the mitochondria, maintaining this transfer by sequentially activating IP3R and MCU appears essential for the survival of DA neurons. The illustration was recreated using Biorender, as described in [158].

**Table 1:** Overview of the DA pathways in PD research. This summarizes key DA pathways, their functions, and relevant studies exploring the pathophysiology, neurodegeneration, and therapeutic strategies in PD.

**Pathway**	**Course and Termination**	**References**
NS pathway	Connects the SN to the dorsal striatum; essential for motor control; severely degenerated in PD	[75-77]
Mesocortical pathway	Projects from the VTA to the prefrontal cortex; implicated in cognition, executive function, and attention	[78-80]
Mesolimbic pathway	Originates in the VTA and projects to the nucleus accumbens; involved in reward, motivation, and impulsivity	[81, 82]
Tuberoinfundibular pathway	Connects the hypothalamus to the pituitary gland; regulates prolactin secretion	[83-85]
Direct and indirect pathways	Modulate basal ganglia output *via* D1 (direct) and D2 (indirect) receptor pathways. Regulated by intracellular signaling cascades (PKA, DARPP-32, ERK, mTORC1)	[61, 86-88]

**Table 2:** Pathogenic mechanisms leading to DA neuronal death.

**Mechanism**	**Description**	**Key Molecules/factors**	**References**
Mitochondrial dysfunction	Impaired mitochondrial respiration leads to neural deficits in energy metabolism	PINK1, Parkin, and complex I	[151]
α-synucleinopathy	Misfolded α-syn aggregates form LBs	LRRK2, α-syn, and ubiquitin	[152]
Neuroinflammation	Chronic glial activation exacerbates neuronal loss	IL-1β, TNF-α, and microglia	[153]
Oxidative stress	Imbalance in ROS production and detoxification damages neurons	NADPH oxidase and glutathione	[89, 154, 155]
Excitotoxicity	Excessive glutamate release leads to Ca^2+^ overload and cell death	NMDA receptors, Ca^2+^, and Glutamate	[156]
Autophagy-lysosomal dysfunction	Impaired degradation of misfolded proteins and organelles	GBA1, TFEB, and LAMP2A	[157]

**Table 3:** Commonly used PD models with mechanism, advantages, and model limitations.

**Model Type**	**Example Model**	**Advantages**	**Limitations**	**Mechanism Simulated**	**References**
Toxin-based	MPTP (mice, primates)	Reproducible DA loss	Limited α-syn pathology	Mitochondrial complex I inhibition	[292]
iPSC-derived	Human iPSC neurons	Human relevance	High cost, technical complexity	Patient-specific mutations	[293]
Toxin-based	6-OHDA (rats)	Rapid onset, localized lesions	Unilateral effects, non-progressive	DA neuron degeneration	[294]
Genetic	LRRK2, Parkin knockout	Links to familial PD	Mild phenotypes	Mitochondrial/autophagic deficits	[295]
Genetic	SNCA overexpression	Mimics LB's pathology	Variable neurodegeneration	α-syn aggregation	[296]

**Table 4:** Cell death pathways with few targeting molecular markers in PD.

**Pathway**	**Description**	**Molecular Markers**	**References**
Apoptosis	Caspase-mediated programmed cell death	Caspase-3, Bcl-2, Cytochrome c	[311]
Ferroptosis	Iron-dependent lipid ROS accumulation	GPX4, lipid ROS, ACSL4	[312]
Autophagy	Self-digestion *via* lysosomal machinery	LC3-II, Beclin-1, p62	[313]

**Table 5:** Neuroprotective strategies under investigation with various mechanisms and a few molecular targets.

**Strategy**	**Mechanism**	**Key Agents**	**References**
Antioxidants	ROS scavenging and mitochondrial support	NAC, vitamin E, CoQ10	[322]
Anti-inflammatory	Suppression of pro-inflammatory cytokines	NSAIDs, TNF-α blockers, minocycline	[323]
Mitochondrial protection	Enhancing ETC and ATP synthesis	MitoQ, nicotinamide, and creatine	[324]
Autophagy/Proteostasis modulation	Boost the protein clearance pathway	Trehalose, rapamycin	[325]
Neurotrophic support	Promotes neuronal survival and growth	GDNF, neurturin, BDNF	[326]

**Table 6:** Emerging gene and cell therapies with target, key genes examples, along with advantages for PD.

**Therapy Type**	**Target/Mechanism**	**Examples/Status**	**Advantages**	**References**
Stem Cell Therapy	Cell replacement with DA neurons	iPSC-derived DA neuron grafts (clinical)	Regenerative potential	[334]
Gene Therapy	Increase DA synthesis or neuroprotection	AADC, GDNF *via* AAV (clinical trials)	local delivery, long-term expression	[335]
RNA-based Therapy	Gene silencing or editing of mutant alleles	ASOs for SNCA/LRRK2, CRISPR-Cas9 (preclinical)	personalized approach, high specificity	[336]

**Table 7:** Overview of PD management strategies with underlying mechanisms and key considerations.

**Strategy Type**	**Specific Approach**	**Mechanism**	**Considerations**	**References**
Pharmacological	Levodopa/Carbidopa	DA replacement	Most effective, long-term motor complications	[341]
Pharmacological	MAO-B inhibitors (*e.g*., selegiline)	Inhibit DA breakdown	Mild symptomatic effect	[342]
Surgical	DBS	Modulates abnormal basal ganglia activity	For advanced PD, careful patient selection is required	[343]
Pharmacological	DA agonists	Stimulate DA receptors	Risk of impulse control disorders	[344]
Lifestyle	Physical therapy, exercise	Enhances mobility and neuroplasticity	Adjunctive, requires motivation	[345]
Experimental	Gene therapy, cell transplantation	Restore functional neurons or gene correction	Early clinical stages, ethical considerations	[346]

## LIST OF ABBREVIATIONS

3-MT: 3-methoxytyramine
AADC: Aromatic L-amino Acid Decarboxylase
AAV: Adeno-associated Virus
ACSL4: Acyl-CoA Synthetase Long-chain Family Member
AD: Alzheimer's Disease
AM: Aminochrome
ATF4: Activating Transcription Factor 4
BBB: Blood-brain-barrier
Ca^2+^: Calcium
CHOP: C/EBP Homologous Protein
CMA: Chaperone-mediated Autophagy
CNS: Central Nervous System
COMT: Catechol-O-methyl Transferase
DA: Dopamine
DAQ: DA Quinones
DAT: Dopamine Transferase
DBS: Deep Brain Stimulation
DOPAC: Dihydroxyphenylacetic Acid
DOQ: DA-o-quinone
ER: Endoplasmic Reticulum
ERAD: ER-associated Degradation
ERK: Extracellular Signal-regulated Kinase
GATs: GABA Uptake Transporters
GBA: Glucocerebrosidase
GC: Glucosylceramide
GDNF: Glial Cell Line-derived Neurotrophic Factor
GPX4: Glutathione Peroxidase 4
GWAS: Genome-wide Association Studies
HVA: Homovanillic Acid
ICD: Impulse Control Disorders
iPSCs: Induced Pluripotent Stem Cells
LBs: Lewy Bodies
LRRK2: Leucine-rich Repeat Kinase 2
MAO-B: Monoamine Oxidase-B
MCU: Mitochondrial Ca^2+^ Uniporter
Mfn: Mitofusin
MOMP: Mitochondrial Outer Membrane Permeabilization
MPTP: 1-methyl-4-phenyl-1,2,3,6-tetrahydropyridine
mTORC1: Mammalian Target of Rapamycin Complex 1
nAChR: Nicotinic Acetylcholine Receptor
NDD: Neurodegenerative Disorder
NIC: Nicotine
NM: Neuromelanin
NS: Nigrostriatal
OMM: Outer Mitochondrial Membrane
PD: Parkinson's Disease
PDD: PD Dementia
PET: Positron Emission Tomography
PFFs: Pre-formed Fibrils
RABs: Ras-Associated Binding Proteins
ROS: Reactive Oxygen Species
SN: Substantia Nigra
SNARE: Soluble N-ethylmaleimide-sensitive factor Attachment Protein Receptor
SPECT: Single-photon Emission Computed Tomography
STN: Subthalamic Nucleus
SVs: Synaptic Vesicles
TH: Tyrosine Hydroxylase
UCHL-1: Ubiquitin Carboxy-terminal Hydrolase L1
UPR: Unfolded Protein Response
UPS: Ubiquitin Proteasome System
VAMP-2: Vesicular Synaptobrevin-2
VMAT2: Vesicular Monoamine Transporter 2
VPS35: Vacuolar Protein Sorting-35
VTA: Ventral Tegmental Area
XBP1: X-box Binding Protein 1
α-syn: α-synuclein
